# ‘Ring’ your future, without changing diaper – Can preventing teenage pregnancy address child marriage in Zambia?

**DOI:** 10.1371/journal.pone.0205523

**Published:** 2018-10-22

**Authors:** J. A. Menon, T. Kusanthan, S. O. C. Mwaba, L. Juanola, M. C. Kok

**Affiliations:** 1 Department of Psychology, University of Zambia, Lusaka, Zambia; 2 Department of Gender Studies, University of Zambia, Lusaka, Zambia; 3 KIT Health, KIT Royal Tropical Institute, Amsterdam, the Netherlands; Aga Khan University, KENYA

## Abstract

Teenage pregnancy and child marriage are prevalent in Zambia and are complexly interrelated issues with common causes and effects. The aim of this study was to explore factors in the social and cultural environment shaping young people’s sexual behaviour, with specific attention to teenage pregnancy and child marriage in Eastern Zambia.

The study was conducted in selected wards in Petauke, Chadiza and Katete districts, using an exploratory mixed-method design including a household survey, focus group discussions and in-depth interviews. The participants included 1,434 young females and males aged 15 to 24, female and male parents and caregivers; grandmothers; traditional leaders; teachers; health and social workers; representatives from youth associations, community-based and non-governmental organizations; and district level policy makers. Qualitative data were analysed using thematic content analysis and NVivo was used to manage the data, while survey data were analysed using Stata.

The study revealed a high prevalence rate of teenage pregnancy (48%) and child marriage (13%) among young women. The mean age at first pregnancy or fatherhood was lower among female (17) than male respondents (20). A clear interlinkage between teenage pregnancy and child marriage was found, the two issues were mutually reinforcing. While teenage pregnancy appeared both as a cause and consequence of child marriage, marriage was mostly a common response to pregnancy. Early sexual debut, limited knowledge and use of contraception, poverty and limited future perspectives as well as sexual and gender norms were identified as the main causative factors of teenage pregnancy and therefore, child marriage.

Based on the findings, a conceptual model to explain the interrelationships between young people’s sexual behaviour, teenage pregnancy and child marriage is discussed. To address teenage pregnancy and child marriage in Eastern Zambia, there is a need to look into the realities and needs of young people regarding sex and relationships.

## Introduction

Teenage pregnancy has been positioned as strong priority in the global health and development agenda. Data indicate that every year 7.3 million teenage girls in developing countries become pregnant. Research has shown that teenage pregnancy leads to major rights violations, especially for young women. Complications from pregnancy and childbirth are among the leading causes of death among girls aged 15–19, teenage pregnancy often limits future perspectives and has also been identified as one of the main reasons for child marriage in many countries [1, 2].

Zambia has a high prevalence of teenage pregnancy. The latest Zambia National Demographic and Health Survey (2013–2014 ZDHS) indicates that 28.5% of young women aged 15–19 had already had a child; a slight increase with the percentage found in 2007 (27.9%) [3]. Evidence shows strong regional differences in the prevalence of teenage pregnancy, with the highest rates in the Western and North Western provinces followed by the Eastern and Southern provinces. Teenage pregnancy rates (15–19 years) were also higher in rural areas (36%) than in urban areas (20%). In addition, teenage pregnancy seems inversely related to educational level and wealth. Twice as many teenagers without education, began childbearing than those with secondary education (53% versus 23%). The percentage of teenagers who began childbearing was highest (45%) among respondents from the lowest wealth quintile; and lowest (10%) among respondents from the highest wealth quintile [3].

Teenage pregnancy can have immediate and lasting consequences for a girl’s health, education and income-earning potential, which is often passed on to her child(ren). Complications from pregnancy and childbirth are among the leading causes of death among girls aged 15–19. Besides high mortality, teenage pregnancy also contributes to illness and disability, related to fistula, complications from unsafe abortion, sexually transmitted infections and HIV [4].

Despite the known negative consequences of teenage pregnancy, economic constraints, socio-cultural and religious norms and practices that impede gender equality or encourage early marriage, early sexual debut, unprotected sex and limited access to sexual and reproductive health (SRH) information and services, including contraceptives, remain barriers to addressing the problems of teenage pregnancy in Zambia [5].

Child marriage, defined as marriage or union under the age of 18, is identified as a key driver of teenage pregnancy. However, pregnancy out of wedlock, poverty, community and household norms and attitudes, low use of contraception and the lack of access to SRH services are all factors that are associated with both child marriage and teenage pregnancy. For example, gender inequality can have an influence on young people’s sexual relationships and their and the family’s choice regarding marriage [6].

A qualitative study carried out in six districts in Zambia, found marriage to be a common response to teenage pregnancy. In many communities, it was the expectation that if a girl becomes pregnant she should marry the father of the child. Marriage, in these cases, was meant to protect the honour of the pregnant girl and her family [7].

Following the above, the SRH of young people received considerable attention over the past years in Zambia. In 2014, the Zambian government introduced comprehensive sexuality education in primary and secondary schools. While the government has specific attention for preventing teenage pregnancy and child marriage, currently, the legal age of marriage at 21 could be lowered to 16 when there is parental consent [8]. Research and experiences from non-governmental organizations (NGOs) show that discussions about sexual health and sexuality are regarded as taboo, especially in rural parts of the country [5]. Rural areas are also characterized by inadequate access to youth friendly SRH services [9]. Therefore, young people in Zambia may not get appropriate guidance on how to avoid pregnancy, while evidence suggests that young people become sexually active between 12 and 19 years [3].

Cultural practices may also shape the sexual behaviour of young people. One of the common cultural practices involving young people is the initiation ceremony or puberty rite. The process of initiating a girl plays a significant role in shaping her perception of sex and sexuality. Initiation rituals are mainly aimed at facilitating young people’s transition into adulthood where topics such as marital roles are addressed including information around sexuality, sexual relations and pleasure. Concerns have been raised about the values inculcated into a woman with regard to sex and sexuality during this rite [10].

Despite this evidence, there is a need for contextualized knowledge on the factors influencing the knowledge and attitudes, and thereby the sexual behaviour of young people in Eastern Zambia. In-depth insights are needed to identify strategies that can address the negative consequences of teenage pregnancy and child marriage. This study aimed at understanding factors in the social and cultural environment shaping young people’s sexual behaviour in Eastern Zambia, with specific attention to teenage pregnancy and child marriage.

## Methodology

This study used a concurrent triangulation mixed-method design collecting in one phase qualitative and quantitative data [11]through household survey, focus group discussions (FGDs) and semi structured, in-depth interviews (IDIs) and key informant interviews (KIIs). Data were integrated in the analysis and interpretation phase and triangulation was used to corroborate and complement findings.

The study was conducted in 2016, in selected rural wards in Petauke, Chadiza and Katete districts, in the Eastern Province of Zambia. The participants included young females and males aged 15 to 24 years; female and male parents and caregivers; grandmothers; traditional leaders; teachers; health and social workers; representatives from youth associations, community-based organizations (CBOs) and non-governmental organizations (NGOs); and district level policy makers (Table 1).

**Table 1:** Study participants.

Method	Types of respondents	Number of respondents
Household survey	Females and Males, 15–24 years	1,434
FGDs	Females 15–19 years	16 (2 groups)
	Males 15–19 years	16 (2 groups)
	Females 20–24 years	16 (2 groups)
	Males 20–24 years	16 (2 groups)
	Parents/ caregivers	16 (2 groups)
IDIs	Females 15–19 years	2
	Males 15–19 years	2
	Females 20–24 years	2
	Males 20–24 years	2
	Parents/ caregivers	2
	Grandmothers	2
	Traditional leaders	2
	Teachers	2
	Health/ social workers	2
	CBO/ youth organization staff	2
KIIs	NGO staff	3
	District policy makers	4

The survey captured respondents’ demographic characteristics and focused on experiences with and perspectives on sexual and reproductive health and rights (SRHR), worries and aspirations, gender, marriage and pregnancy (S1 File). Sample size calculations were based on being able to detect a 10% reduction over a 5-year period in the percentage of women aged 15–24 who have had a live birth or who were pregnant with their first child. The percentage found in the 2013–2014 ZDHS (CSO, 2014) was 53% for Nyimba, a neighbouring district–and was taken because it presented a “worst case scenario”. This provided a sample size of 391 for females (pw = 0.8; sig<0.05). Taking into account possible “design effects” because of the clustered sampling, this was multiplied by 1.5 (yielding 578 females). On top of this, 130 males were added, to gather for a 75%-25% selection of females and males. The total sample size (717) was multiplied by two, to be able to detect changes in teenage pregnancy rate between an intervention area (Chadiza and Petauke) and a control area (Katete), related to a multicomponent intervention implemented by the Yes I Do Alliance (2016–2020), aiming at reducing teenage pregnancy and child marriage. However, in this article, no distinctions are made for intervention and control areas. Results from implementation and control area will be assessed against each other in 2020.

The sampling frame was drawn from the administrative demarcations of the country, which is the census frame. The sample was selected in two stages. First, 64 standard enumeration areas (SEAs) were semi-randomly selected with probability proportional to size. (In Chadiza and Petauke, the districts were interventions were to be launched, a semi-random selection of SEAs from specific wards in which the interventions were to be conducted was done. Therefore, SEAs were randomly selected from an a priori defined number of wards in the districts. In Katete, a full random selection of SEAs and respondents was done.) In the second stage, in all three districts, 25 households were randomly selected in each of the SEAs by applying a fixed interval based on the listing of households per SEA. Upon arrival at the household, young people were randomly selected, but with taking into account the sex and age of the potential respondent. If an eligible respondent was not found, the next household was approached.

The qualitative study component was only conducted in Chadiza and Petauke. Participants were asked about socio-cultural norms and values around gender and youth sexuality, the causes and consequences of child marriage and teenage pregnancy, changes in experiences, feelings and opinions over time, and if interventions were working or not. Selection of participants was purposeful, based on obtaining information rich cases, and recruitment was facilitated by village leaders. The survey questionnaire and FGD and interview topic guides were translated in Chewa and after a training, data collectors pre-tested all tools to identify necessary adjustment in terms of language, length and reactions to sensitive issues. The pre-test was conducted in a district different from the three study districts, however, it was ensured that the (rural) setting was comparable to that in the study districts. Results of the pre-test were not included in the analysis of the overall results. Daily debriefing sessions with all data collectors were held to discuss key findings, refine lines of inquiry and identify saturation of themes.

Quantitative data were analysed in STATA 13 through descriptive statistics of demographic and behavioural data disaggregated by sex, age and marital status where relevant. Interviews and FGDs were digitally recorded, transcribed and, where applicable, at the same time translated into English and independently checked by someone not involved in transcribing. Content analysis of qualitative data was carried out using a comprehensive thematic matrix, based on the topic guides. We used NVivo version 11 to code all transcripts according to the matrix, and write narratives per theme that facilitated the triangulation with the quantitative data.

Young people’s sexual behaviour and the factors that influence it emerged as an important theme in the overall study. As expected, they influenced the situation around teenage pregnancy and child marriage in the communities. Therefore, both quantitative and qualitative data related to these themes were analysed in-depth, focusing on: characteristics on young people’s sexual behaviour and the factors influencing it. With respect to the latter, an inductive analysis yielded three major themes, which are presented in the results section and further discussed through a proposed conceptual model.

The study was approved by the Royal Tropical Institute Ethical Review Committee in Amsterdam and the Humanities and Social Sciences Research Ethics Committee at the University of Zambia, and further permission was obtained from the Ministry of Health. Informed consent was obtained from all participants and in the case of minors (children below the age of 16), consent was sought from parents or guardians. Voluntary participation and confidentiality were upheld. The respondents of the survey were compensated with a refreshment allowance, because of the length of the questionnaire, and the participants of FGDs were reimbursed with a transport allowance, in accordance with the national policy.

## Results

The results are presented in four main sections. First, data on demographics of the survey respondents are presented to contextualize the findings. The second section is about the prevalence of teenage pregnancy and the interlinkages that were found with child marriage. After that, the main characteristics of young people’s sexual behaviour in the study areas are described. Finally, factors that were found to influence young people’s sexual behaviour are presented.

### Demographic characteristics of survey respondents

Most respondents lived in the district where they were born and raised. The majority of the surveyed young people were not married; there were more married young women (22%) than married young men (8%). The proportion of married young people was higher among those aged 18 to 24 (37% of the females and 13% of the males) than among those aged 15 to 17 (3% of the females and no males). Most respondents had primary or secondary education levels with very few cases of tertiary education. Most young women and men were unemployed without own income. Fathers were reported to be the primary income earners (52%). Most parents had some kind of education. About one quarter of the participants’ mothers and one third of their fathers had received no education (Table 2).

**Table 2:** Background characteristics of the survey participants.

Background variables	Females (n = 1,006)	Males (n = 449)
Age (n = 1,445)		
15–17	45.4%	33.1%
18–24	54.7%	66.9%
Marital status (n = 1,445)		
Not married	78.4%	91.5%
Married	21.6%	8.5%
Current educational level (n = 1,445)		
Completed	3.0%	7.3%
Other	33.8%	26.3%
Primary	37.0%	37.0%
Secondary	25.6%	28.0%
Tertiary	0.3%	1.0%
Vocational	0.2%	0.3%
Employment status (n = 1,445)		
Employed	13.4%	30.7%
Not employed	86.6%	69.3%
Father’s education (n = 1,445)		
No education	25.2%	26.8%
Primary	20.3%	19.2%
Secondary	17.3%	24.6%
Tertiary	2.2%	3.4%
Unknown	33.9%	25.4%
Vocational	1.1%	0.7%
Mother’s education (n = 1,445)		
No education	37.5%	33.9%
Primary	25.7%	24.3%
Secondary	25.1%	20.1%
Tertiary	0.7%	1.6%
Unknown	22.2%	20.1%
Vocational	0.3%	0.0%

### Teenage pregnancy and child marriage: Prevalence and interlinkages

#### High prevalence of teenage pregnancy and child marriage among young women

The study revealed a high prevalence of teenage pregnancy and child marriage in Eastern Zambia, particularly among young women. In relation to teenage pregnancy, the mean age at first pregnancy was lower among female respondents (17) than among male respondents (20) who had impregnated a girl. Among all respondents, 31% of the females already had a child against 10% of the males.

The teenage pregnancy rate among female respondents aged 20–24 was 48%. In addition, more than half of all young women who had had a teenage pregnancy (67%), reported that they did not desire to become a mother at that time, suggesting that most teenage pregnancies were unplanned.

Furthermore, a child marriage rate of 13% among female respondents aged 18–24 was found, while among male respondents in the same age range, only one case of child marriage was found (0.3%) (Table 3).

**Table 3:** Prevalence of teenage pregnancy and child marriage in Eastern Zambia.

Teenage pregnancy % (n)		Child marriage % (n)	
Women aged 20–24 who had their first child under the age of 20 (teenage pregnancy rate)	47.7% (298)	Women aged 18–24 who were married or in a union before the age of 18 (child marriage rate)	13.2% (552)
Men aged 20–24 who had their first child under the age of 20	1.0% (482)	Men aged 18–24 who were married or in a union before the age of 18	0.3% (301)
Women aged 15–24 who had a child	31.0% (1,006)	Women aged 15–24 who were married	18.5% (1,006)
Men aged 15–24 who had a child	9.6% (448)	Men aged 15–24 who were married	8.0% (449)
Mean age at first pregnancy among young women aged 15–24	17.5 (min = 12, max = 23)	Mean age at marriage among young women aged 15–24	17.9 (min = 13, max = 24)
Mean age at the first born child among young men aged 15–24	20.4 (min = 16, max = 24)	Mean age at marriage among young men aged 15–25	20.4 (min = 17, max = 24)

#### Interlinkages between teenage pregnancy and child marriage

The data indicate a significant interlinkage between teenage pregnancy and child marriage. Teenage pregnancy appeared both as a cause and as a consequence of child marriage. Some participants mentioned teenage pregnancy and particularly delivery complications as a negative consequence of child marriage. However, as illustrated in the following excerpt from an interview with a young woman, teenage pregnancy was more commonly highlighted as a driver and reason for child marriage.

*Interviewer*: *When a girl is 15 and becomes pregnant what will happen to her let’s say she is in school?*

*Participant*: *She will drop out of school and the parents will send her into early marriage. (IDI, Girl 16 years)*

**Fig 1:**
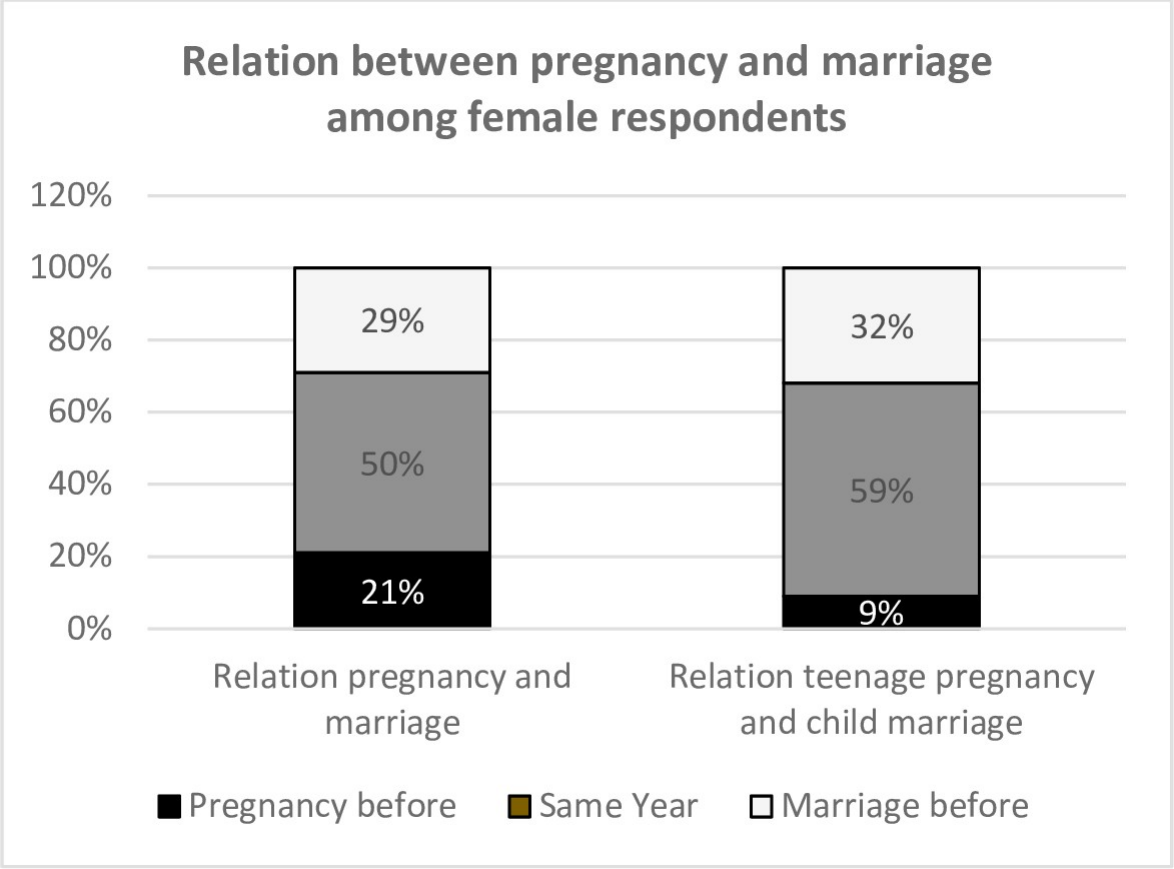
Pregnancy and marriage: What came first?.

Survey data also indicate that marriage generally occurred later than the pregnancy. Among all female respondents of the sample who had already had a child and were married, 21% had the pregnancy before the marriage and 50% reported the same year for both. When looking specifically only at the cases of child marriage, 59% had a teenage pregnancy in the same year as the marriage, and 9% before the marriage (Fig 1). Considering that when the occurrence of the first pregnancy and of marriage were in the same year, it was likely, as reported in the FGDs and interviews, that the pregnancy was the reason for marriage. The average age gap between married girl and boy was four years, showing that marriage was mainly between age mates.

#### Child marriage as a response to teenage pregnancy

The study found that marriage decisions in cases of teenage pregnancy were often taken or influenced by parents. As one young man stated during one of the interviews: *‘when a boy makes a girl pregnant*, *the parents will ask them to marry’*.

According to participants’ accounts, usually the parents of the young woman asked the impregnator to take responsibility for the pregnancy. Marriage was seen as the main means through which the man could take responsibility for the pregnancy, and economic reasons were central to this decision. First, a pregnant and unmarried young woman represented an additional burden for her family and therefore, transferring the responsibility to a husband eased the burden. Second, with a marriage, the family of the young woman received a bride price in form of money or cattle. Hence, in cases of pregnancy, to lessen the burden and receive an income, marriage was seen as a solution.

*She will give problems to the parents because now it’s another burden to the parents to be caring for her and as well as the baby*. *And parents may insist that the boy who impregnated her should marry her. (IDI, Traditional leader)*

In addition, marriage would also prevent shame and another pregnancy outside wedlock. Participants argued that young women often agreed to marry as it was seen as the next logical step. Even more, participants´ narratives suggest that limited future perspectives in terms of education and jobs influenced the perception of marriage as the best alternative. In cases where the young man refused to marry, the young woman’s parents charged an amount as a way to compensate for the consequences of the pregnancy in the young woman’s and their life such as financial difficulties and limited future opportunities due to school drop-out This evidences the centrality of economic reasons to marry in case of pregnancy. This is illustrated in the following quote from an FDG with caregivers.

*When a girl is pregnant and you the man responsible refuses, parents of the girl will charge you even K4,000*.

*The reason they charge so much is that they feel bad that the future of their girl child has been destroyed even after spending so much money on her and also making her become a mother at a tender age*.

### Young people’s sexual behaviour

Teenage pregnancy is an evidence of young people’s sexual activity. The study found three main aspects characterizing young people’s sexual behaviour in Eastern Zambia: early sexual debut, relative knowledge about SRHR, and low use of contraceptives.

#### Early sexual debut

The study reveals that pre-marital sexual relations were common, and that teenage girls and boys were generally sexually active. Participants highlighted early sexual debut as a key driver of teenage pregnancy and argued that sexual debut occurred at ages as early as 9 and 10 years. Social and gender norms seemed to influence young people´s behaviour. Participants referred to the dressing, behaviour, and look as ‘signs’ that young women have started having sexual relations.

*Just like my friend has said we look at their dressing and behaviour then you can tell that this girl has started sleeping with boys*. *(FGD, Girl 17 years)*

At the same time, the quantitative data show a mean age of first pregnancy at 17 years, with the earliest case being at the age of 12. This suggests that young people are sexually active before they marry, but probably not as early as at ages of 9 or 10 years.

#### Limited knowledge and use of contraceptives

**Fig 2:**
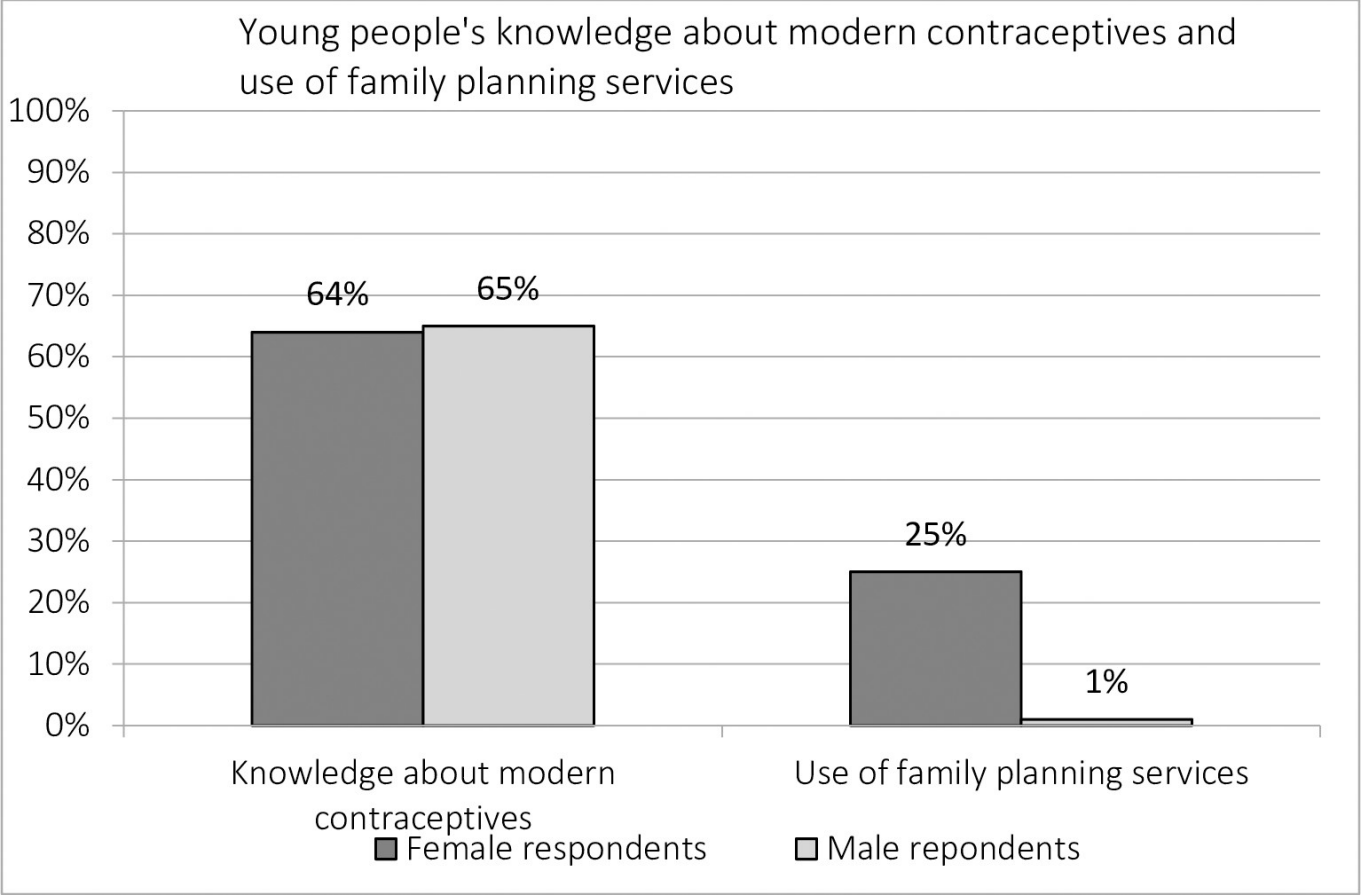
Young people’s knowledge and use of contraceptives.

The study found that young women and men did have knowledge about SRHR. More than half of the respondents (66%) reported having ever received any form of sexuality or sexual health education. Radio was reported to be the main media source from which young people got information (58%), followed by television (30%). Furthermore, teachers were mentioned most as the source of SRHR education (48%), followed by health providers (38%) and friends (27%). Only 19% reported to have received SRHR education from parents. Among all female and male respondents, 64% had knowledge about modern contraception and 88% said they know how to prevent a pregnancy (Fig 2). While abstinence was the most mentioned method to prevent a pregnancy (75%), condoms where mentioned by 56% of the respondents.

However, although most young people seemed to know how to prevent a pregnancy and showed awareness of the negative consequences of teenage pregnancy, nearly half of the respondents (45%) reported being worried about early pregnancy. Moreover, despite young people’s general knowledge about contraceptives, the use was low among all respondents; 18% reported having ever used family planning services, 30% reported to have ever used contraceptives and 21% reported to currently use them, with injections being the most popular method.

Young people´s sexual behaviour, generally characterized by early sexual debut and low use of contraceptives, explains why the teenage pregnancy rate was high. At the same time, the findings on young people´s sexual behaviour indicate that the low use of contraceptives was not always due to a lack of information, because participants showed relative knowledge on modern contraceptives, and high awareness on how to prevent pregnancy. Still, most teenage pregnancies were unplanned.

### Factors shaping young people’s sexual behaviour

To better understand the drivers of teenage pregnancy, the study explored the factors in the social and cultural environment that could shape young people´s sexual behaviour. It was found that several factors simultaneously influenced young people’s sexual behaviour, and these can be summed and grouped in three main categories: a) poverty and limited future perspectives, b) social, gender and sexual norms; and c) availability of SRH services and information.

### Poverty and limited future perspectives

Participants’ narratives suggest that poverty and a lack of future perspectives was influencing young people’s sexual debut. Transactional sex was commonly mentioned to happen in young women’s relations with men. Various participants argued that young women from low income households engaged in sexual relations with boys and men in exchange for money to lessen the household economic burden. Others referred to sex as a means used by young women to get other goods such as clothes.

*Some where they come from are very poor*. *They start sleeping around with men in exchange for money to help at home (IDI, Girl 18 years)*

*They start at 12 because maybe she will want a sweet or something nice and doesn’t have money*. *The boy will start buying the things she wants when he asks to have sex with her she will fail to refuse (IDI, Girl 16 years)*

### Socio-cultural, gender and sexual norms

Young people’s sexual behaviour was influenced by multiple and interlinked social, gender and sexual norms, such as perceptions around childhood and adulthood, sexual moralities or gender identities and roles.

Initiation ceremonies seemed to play a key role in the transference of these norms, especially around sexuality and gender roles. Young women learned about marriage and were taught how to take care of their husbands and house. Participants related these ceremonies to a symbolic entrance of adulthood, which in turn, leads to adulthood behaviour such as active sexuality. These ceremonies take place when young women hit puberty, which corresponds to the age that most respondents (63%) reported as the perceived age at which girls become adults which is between 15 and 20 years. As the following quotes illustrate, both female and male participants mentioned that initiation ceremonies incentivise young people´s curiosity for sex and engaging in sexual relationships.

*“For girls because of the tradition that they are taught when they become of age they want to go and try it out that is why they start having sexual relations at an early age*.*”* (FDG, Girl 18 years)

*“It’s the traditional ceremonies that influence these children*, *because after they are taught they want to experiment*.*”* (FDG, Boy 20 years).

Data reveal that it was a prevailing that sexual relations should take place within marriage. That explains why abstinence was the most commonly reported method to prevent pregnancy by young people. As mentioned before, there seemed to be a double standard in relation to sexual norms. Half of the respondents (50%) disagreed with the statement that is it appropriate for a young woman to propose condom use, while more than three quarters of all respondents (80%) agreed that it was easy for young men to propose the use of a condom. In a similar line, more male (85%) than female respondents (62%) reported feeling confident in insisting on the use of a condom.

Gender based violence did not appear as a main driver of teenage pregnancy, however, few study participants mentioned sexual violence resulting in teenage pregnancy–and in some instances, teachers were involved. Eighty-three percent (83%) of the female respondents reported to have never experienced sexual harassment, while 7% reported to experience it less than once a month, 5% once or twice a month, and 0.6% every day.

### Access to SRH information and services

When asked about the preferred source of SRHR information, while in the survey young people mostly mentioned health facilities and schools, in the FGDs and interviews many young people said they were most comfortable in approaching their grandparents for advice and information. As the following quote illustrates, young people seemed to view their parents as authoritative; they felt shy to discuss sensitive topics with them and considered it inappropriate.

*“Because it is easy to communicate with your grandparents than it is with your parents*, *because with your parents you may be shy*, *but with your grandparents you can say anything you have in mind*.*”* (FDG Young man 20–23 years)

**Fig 3:**
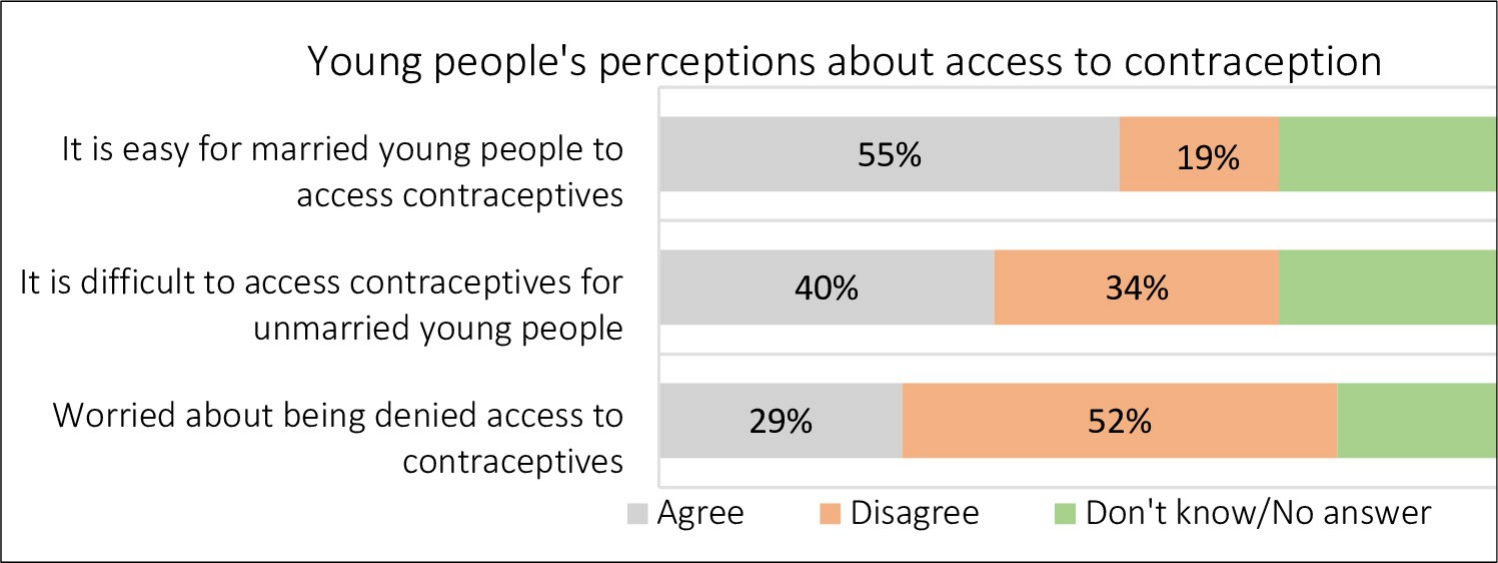
Young people’s perceptions on access to contraception.

It seemed that formal agents and sources of information and services were often not available or effective in reaching out to the youth. The data reveal different opinions of young people about the access to contraceptives. About 39% of females and 40% of males agreed with the statement that contraceptives were difficult to access in the community; and 34% of females and 33% of males disagreed with the statement that contraceptives were difficult to access. About half of the respondents (49% of the females and 55% of the males) reported not being worried about being denied access to contraceptives. However, access to contraceptives was reported to be easier for married young people than for the unmarried ones. About 40% of the female and male respondents agreed that it was difficult for unmarried young people to access contraceptives, and 55% agreed that it was easy for married young people to access contraceptives (Fig 3).

In relation to the use of SRH services, more than half of the respondents had ever used any SRH service. About 58% of the female respondents reported having utilised SRH services, compared to 40% of the male respondents. Among female respondents, antenatal care was the main used SRH service (32%) followed by family planning (25%).

## Discussion

**Fig 4:**
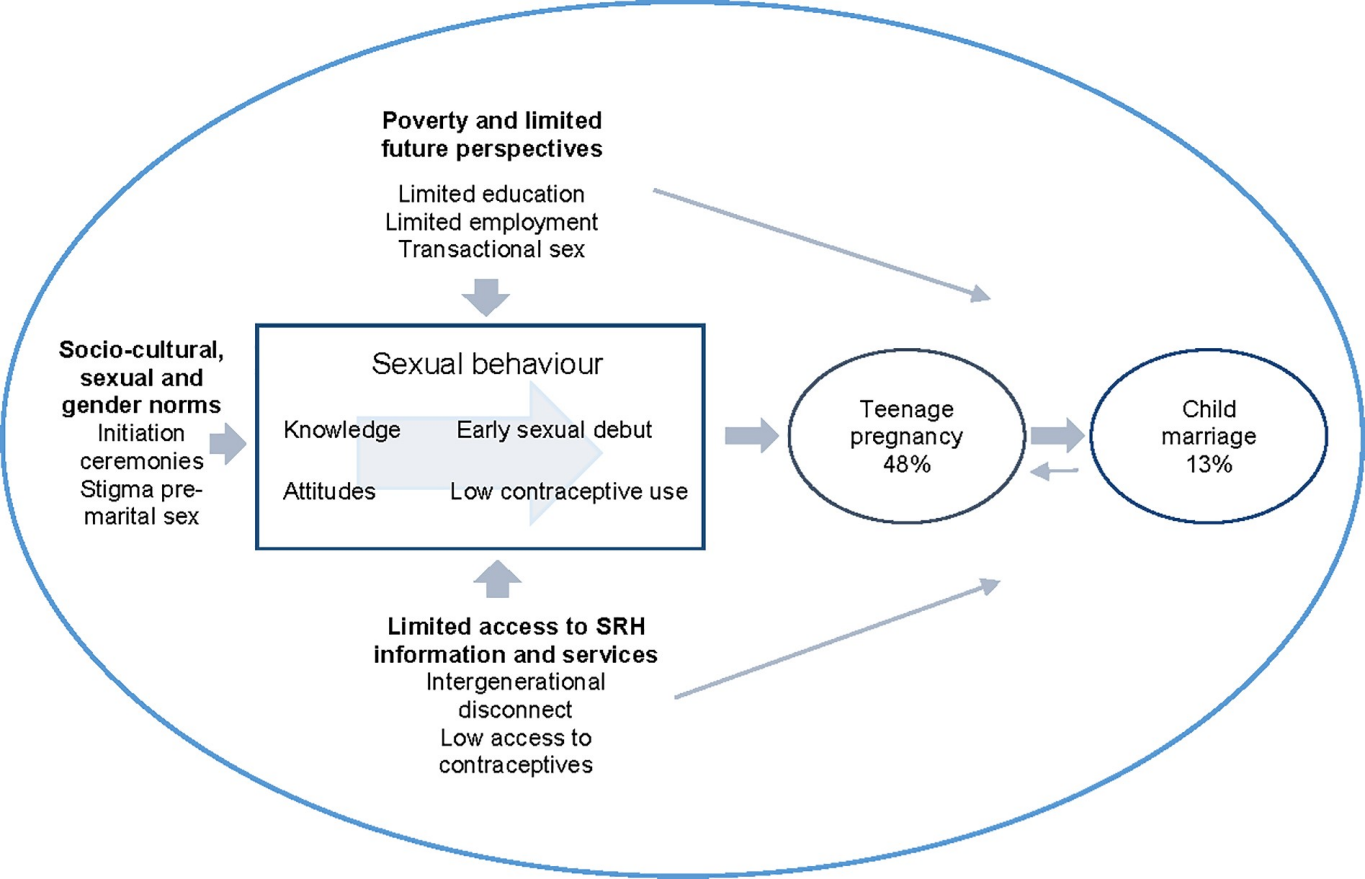
Factors influencing high risk sexual behaviour leading to teenage pregnancy and culminating in child marriage in the Eastern province of Zambia.

This study aimed to understand factors in the social and cultural environment shaping young people’s sexual behaviour, with specific attention to teenage pregnancy and child marriage in selected districts in Eastern Zambia. Based on our findings, we propose the following conceptual model (Fig 4):

The model summarizes the three main findings of the study. First, that in Eastern Zambia teenage pregnancy was more prevalent than child marriage and often preceding child marriage. Second, teenage pregnancy was a direct result of young people’s sexual behaviour which was characterized by early sexual debut, and a gap between a general knowledge on contraceptives and use of it. Third, young people’s sexual behaviour, determined by their knowledge and attitudes, was influenced by various contextual factors which included poverty and limited future perspectives, sexual and gender norms, and access to SRH information and services.

Although all elements presented in the model are interrelated and often mutually reinforcing each other, the model visualizes the main relations found in this study in Eastern Zambia, highlighting the centrality of young people’s sexual behaviour to address teenage pregnancy and child marriage.

### Teenage pregnancy as a driver of child marriage

The study found that the overall prevalence of teenage pregnancy and child marriage in the study population was high among young people in Eastern Zambia. The teenage pregnancy was higher than the child marriage rate, with more females than males becoming a parent at a young age. Child marriage was also related to gender, women married younger than men, but marriage was mainly between age mates, which corroborated the centrality of young people’s sexual behaviour and the importance in understanding it better. These results are in line with other studies [3, 12, 13].

Studies conducted in urban communities of Kenya have shown that poverty is associated with early sexual engagement [14, 15]. The current study also supports the findings of previous scholars regarding the association between early sexual debut and teenage pregnancy. A study from Ethiopia concluded that a growing number of young people become sexually active before marriage and as a consequence, the rate of unplanned pregnancies among this group, particularly among those with unmet need for contraceptives, increases [16].

### Factors influencing young people’s sexual behaviour

Young people’s sexual behaviour was characterized by early sexual debut. The study found that the mean age at first pregnancy was lower among female respondents (17 years) than the age at which male respondents became father (20 years). This might be as a result of differences in timing of sexual maturity between girls and boys. Generally, females mature earlier than males [17]. Furthermore, there was a gap between knowledge and use of contraceptives. Access to contraceptives seemed limited. A systematic review on factors influencing contraceptive use in sub-Saharan African found that low contraceptive use is associated with misconceptions on side-effects, male partner disapproval, socio-cultural norms about fertility, a lower education level and unemployment [18], part of these factors were found in this study as well.

### Poverty and lack of future perspectives

Poverty and a lack of future perspectives were influencing young people’s sexual behaviour, leading to teenage pregnancy and child marriage. Several studies attest to poverty being one of the critical factors which contributes to child marriage. They observe that young girls in poor families are often regarded as an economic burden, while marrying them to older men is believed to bring social as well as financial benefits [12, 19]. Where girls are viewed as additional burden on family resources, they tend to be married off earlier as a family survival strategy [20, 21]. A recent cross sectional study from South Africa also highlights poverty and low socio-economic status as a cause of child marriage [22].

In our study, most girls did not marry older men, however, shifting the financial responsibility for the girl to a (future) husband was found to be important. In some societies, girls are regarded as a commodity that can be traded to settle disputes or debts [23]. This phenomenon was not found to take place in Petauke, Chadiza and Katete districts in Eastern Zambia.

Besides economic factors (directly) leading to child marriage, we found that economic hardship had an effect on young people’s sexual behaviour, leading to teenage pregnancy and later on, marriage. A study using Demographic and Health Survey data in sub-Saharan Africa confirms that community poverty is associated with teenage pregnancy [24]. Transactional sex was found to take place frequently. In our conceptual model, it is related to poverty and limited future perspectives, however, research from Malawi has shown that young women’s aspirations to social mobility, economic independence and joy from goods such as clothes and lotion play a role. Furthermore, transactional sex is related to establishing, maintaining and sustaining ties binding women and men in social relationships, and as such can be seen as an issue related to socio-cultural norms as well [25].

### Socio-cultural, gender and sexual norms

The study revealed that socio-cultural factors, gender and sexual norms had a strong influence on adolescent sexual behaviour which in turn fuelled teenage pregnancy and child marriage.

Initiation ceremonies upon puberty and role expectations of a girl child who has been initiated into adulthood were among such factors. These findings confirm previous research in sub-Saharan Africa which found that initiation ceremonies facilitate early sexual experimentation among adolescents [26]. Some traditional practices like initiation ceremonies such as ‘Chinamwali and Nyau’ have been attributed to the high prevalence of child marriages in Zambia, particularly in the Eastern Province [27]. Whereas it is acknowledged that initiation ceremonies are part of ‘passage’ rites and are an integral part of traditional society, there is evidence of traditional instructors adopting a more pragmatic approach of imparting economic empowering skills as well as self-assertive behaviours, that will help the young people on how to stand for themselves in (sexual) relationships [9].

While the stigma related to pre-marital sex was there in the communities, young people were found to engage in sexual relationships outside marriage. This behaviour, being not in line with the norm, could lead to child marriage. This and previous research has shown that child marriage is considered by many to be a way to protect young girls from pregnancy out of wedlock which is associated with shame. Yet, early marriages leave them physically and socially vulnerable to gender inequality, illness, poverty, and violence [28].

### Limited access to sexual and reproductive health information and services

It was found that young men and women had relatively limited accurate knowledge about SRH, despite having available sources of information such as the media (radio and television), schools and health facilities. Research has shown that sexuality education helps in developing skills in making informed choices among young people [29]. Our findings suggest the need for more effective and comprehensive sexuality education for young people in eastern Zambia, also in their family and community setting, which would require more effective inter-generational communication.

The results of this study also suggest that SRH services were not always available or effective in reaching out to the youth. Access to contraceptives was easier for married young people than for the unmarried. The World Health Organization (WHO) also observed that use of any contraceptive method among unmarried adolescents is lowest in African countries [30]. Similarly, previous research has consistently shown that many adolescents in sub-Saharan African countries underuse SRH services due to barriers such as service costs and distance, lack of awareness about where to get contraceptives and treatment for sexually transmitted infections, embarrassment, lack of confidentiality and privacy, and negative provider attitudes [31–33]. The current health care service provision set-up seems to be little attractive to young people. Some common reasons for this shown by other studies include lack of privacy and few trained health workers that can provide effective adolescent friendly services [34, 35].

### Study limitations and recommendations for future research

Study findings are based on self-reported experiences, opinions and perceptions of young people and other stakeholders. The use of mixed methods and trained data collectors reduced the probability of getting social desirable answers. This study looked at factors in the social and cultural environment shaping young people’s sexual behaviour, as this was a profound emerging theme in a broader baseline study on teenage pregnancy and child marriage. The conceptual model we propose provides insights into the relationships between our major findings, but further research is needed to refine it and look into nuances related to factors influencing young people’s sexual behaviour in this context. For example, specific interventions that are gender sensitive ought to be paid attention to by future researchers.

## Conclusion

This study has shown that teenage pregnancy and child marriage are experienced at worryingly high rates in Petauke, Katete and Chadiza of the Eastern Province of Zambia. The problems are mutually reinforcing, with teenage pregnancy often being a precursor to child marriage. It can be concluded that teenage pregnancy and child marriage are caused by a multiplicity of factors emanating from socio-cultural factors to limited access to SRH information and services and high poverty levels and limited future perspectives of the young people. In light of the obtaining situation of the young people of Petauke, Chadiza and Katete, the government, together with cooperating social partners and the youth themselves are prodded to double their efforts in challenging social and cultural norms, creating educational and economic opportunities and improving access to information and services.

## Supporting information

S1 File Survey questionnaire. Survey questionnaire used, English version page 1–51; Chewa version page 51–105.
(PDF)
