# Supplementary material for: ‘Ring’ your future, without changing diaper – Can preventing teenage pregnancy address child marriage in Zambia?
Source: PLoS One. 2018 Oct 22;13(10):e0205523. doi: 10.1371/journal.pone.0205523 (PMC6197635; doi:10.1371/journal.pone.0205523)
Supplement: S1 File — Survey questionnaire used, English version page 1–51; Chewa version page 51–105. (PDF) [file pone.0205523.s001.pdf]

ODKSurvey_Zambia_FINAL

Section 0 - General Information

0,01 Please register the location

latitude (x.y °) longitude (x.y °) altitude (m) accuracy (m)

0,02 In which district are you located?

required

- (●) Chadiza
- ( ) Petauke
- ( ) Katete

0,02z1 In which ward are you located?

- ( ) Chanjowe
- ( ) Chilenga
- ( ) Kabvumo
- ( ) Kandabwako
- ( ) Kapachi
- ( ) Mangwe
- ( ) Manje
- ( ) Naviluri
- ( ) Nkhumba
- ( ) Nsadzu
- ( ) Taferansoni

0,02z2 In which ward are you located?

- ◯ Chalimanyama
- ◯ Kovyane
- ◯ Mateyo Mzeka
- ◯ Mawanda
- ◯ Mbala
- ◯ Msumbazi
- ◯ Nsimbo
- ◯ Nyakawise
- ◯ Nyika
- ◯ Ongolwe
- ◯ Singozi
- ◯ Ukwimi

0,02z3 In which ward are you located?

- ◯ Chavuka
- ◯ Chimtende
- ◯ Chitawe
- ◯ Chiwuyu
- ◯ Dole
- ◯ Kadula
- ◯ Kafumbwe
- ◯ Kapangulula
- ◯ Kapoche
- ◯ Katiula
- ◯ Mkaika
- ◯ Mngo'mba
- ◯ Mnyamazi
- ◯ Mphangwe
- ◯ Mwandafisi
- ◯ Nyamasonkho
- ◯ Sinda
- ◯ Vulamkoko

0,02z4 In which CSA are you located?

0,02z5 In which SEA are you located?

0,03 Ask for consent to take part in the interview

Please explain the objective and consequences of participation to this survey to the respondent. If minor, has the main caregiver/ guardian of the family provided consent for the interview?

- Consent
- Refused

0,04 Why did the person refuse to participate?

0,05 Interviewers name

- Candy Kunda
- Cynthia Simakoloyi
- Glenda Chileshe
- Grace Nalikando
- Helen Mtonga
- Isaac Jerry
- Mable Maboshe
- Mabvuto Tembo
- Musonda Mwape
- Nguli Zulu
- Pungashi Manda
- Raquel Kazembe
- Sarah Kamanga
- Sarah Mbobola
- Sheila Khonje
- Yvone Chileshe

Section 1 - Respondents background

1,01 Sex of respondent

- Female

- ◯ Male

1,02 How old are you now?

- ◯ Years
- ◯ Don't know
- ◯ No Answer

1,03 Age in numbers

____

1,04 Do you have a national ID card or birth registration card?
Inde| Ai | SD = Sindidziwa | PY = Palibe yankho

- ◯ Yes
- ◯ No
- ◯ Don't know
- ◯ No answer

1,05 Do you belong to any religion?
Inde| Ai | SD = Sindidziwa | PY = Palibe yankho

- ◯ Yes
- ◯ No
- ◯ Don't know
- ◯ No answer

1,06 Which one?

- African methodist
- UCZ
- 7th day adventist
- Reformed church
- Baptist
- New apostolic
- CMML
- Salvation army
- Jehovah's witness (watchtower)
- Pentecostal
- Presbyterian
- Anglican
- Catholic
- Muslim
- Hindu
- Other

Specify other.

1,07 Which tribe do you belong to?

- ◯ Bemba
- ◯ Lozi
- ◯ Tonga
- ◯ Kaonde
- ◯ Lunda
- ◯ Luvale
- ◯ Other

Specify other.

___

1,08 Who do you live with?
You may mark more than 1 option

- [ ] Mother
- [ ] Father
- [ ] Mother in law
- [ ] Father in law
- [ ] Grandmother
- [ ] Grandfather
- [ ] Aunt
- [ ] Uncle
- [ ] Sister(s)
- [ ] Brother(s)
- [ ] With wife/husband
- [ ] Alone
- [ ] Other

Specify other.

____

1,09 Total size of household
This should be the total number of people who eat together/ live together as a family unit. Write down "1" if respondent lives alone.

____

1,10 What type of education have you received?
Please mark only one option

- ◯ No education
- ◯ Junior primary
- ◯ Senior primary
- ◯ Junior secondary
- ◯ Senior secondary
- ◯ Vocational training
- ◯ Tertiary education

1,10z Which type of school?

- ◯ Government
- ◯ Private
- ◯ Other

Specify other.

____

1,11 How many years of school have you completed?
Number of years starting from primary school, do not count repeated years

____

1,12 What is the level of education you are currently studying?

Please mark only one option

- ◯ Junior primary
- ◯ Senior primary
- ◯ Junior secondary
- ◯ Senior secondary
- ◯ Vocational training
- ◯ Tertiary education
- ◯ Completed
- ◯ Other

Specify other.

____

1,13 Have you ever dropped out of school and for what reason?

Do not read the list, tick all that apply

- [ ] No
- [ ] Lack of fees/materials
- [ ] Domestic/family responsibility
- [ ] Illness
- [ ] Parents died while still young
- [ ] Pregnancy/got a child
- [ ] School too far/not accessible
- [ ] No time
- [ ] Did not like school
- [ ] Got married
- [ ] Suspended/expelled
- [ ] Feared discrimination/stigma
- [ ] Don’t remember
- [ ] No answer
- [ ] Other

Specify other.

____

1,14 What type of education has your mother received?

- ◯ No education
- ◯ Junior primary
- ◯ Senior primary
- ◯ Junior secondary
- ◯ Senior secondary
- ◯ Vocational training
- ◯ Tertiary education
- ◯ I don't know

1,15 How many years of education has she completed?
Number of years starting from primary school. IF RESPONDENT DOES NOT KNOW TYPE 'don’t know'

____

1,16 What type of education has your father received?

- ◯ No education
- ◯ Junior primary
- ◯ Senior primary
- ◯ Junior secondary
- ◯ Senior secondary
- ◯ Vocational training
- ◯ Tertiary education
- ◯ I don't know

1,17 How many years of education has he completed?
Number of years starting from primary school. IF RESPONDENT DOES NOT KNOW TYPE 'don't know'

____

1,18 Have you received any income in the last six months?
Inde| Ai | SD = Sindidziwa | PY = Palibe yankho

- ◯ Yes
- ◯ No
- ◯ Don't know
- ◯ No answer

1,19 From whom or where did you receive it?
You may mark more than 1 option

- ☐ Mother
- ☐ Father
- ☐ Aunt
- ☐ Uncle
- ☐ Sister
- ☐ Brother
- ☐ Boy-girlfriend/spouse
- ☐ Own income: temporary job
- ☐ Own income: permanent job
- ☐ Own business
- ☐ Government/ social assistance
- ☐ Other

Specify other.

____

1,20 Employment status

Do not read the list, tick all that apply

- ☐ Unemployed
- ☐ Unpaid work (e.g. homemaker/housewife)
- ☐ Subsistence farming
- ☐ Informal trading
- ☐ Casual, daily laborer
- ☐ Contract work
- ☐ Self-employed
- ☐ Part-time permanent salaried employment
- ☐ Full-time permanent salaried employment

1,21 What type of work do you do?
Do not read the list, tick all that apply

- ☐ Agriculturalist
- ☐ Pastoralist
- ☐ Petty trading
- ☐ Small business owner
- ☐ Household help
- ☐ Casual labor
- ☐ Construction
- ☐ Factory work
- ☐ Garment worker
- ☐ Wholesale and retail
- ☐ Hotels and restaurants
- ☐ Transport
- ☐ Real estate activities and rental
- ☐ Remittances (from abroad)
- ☐ Government employee
- ☐ NGO
- ☐ Other

Specify other.

1,22 What are your household's primary sources of income?

Do not read the list, tick all that apply

- [ ] Agriculturalist
- [ ] Pastoralist
- [ ] Petty trading
- [ ] Small business owner
- [ ] Household help
- [ ] Casual labor
- [ ] Construction
- [ ] Factory work
- [ ] Garment worker
- [ ] Wholesale and retail
- [ ] Hotels and restaurants
- [ ] Transport
- [ ] Real estate activities and rental
- [ ] Remittances (from abroad)
- [ ] Government employee
- [ ] NGO
- [ ] Other

Specify other.

1,23 How much money (income) comes into your household each month (on average)?
Write income as reported. Young respondents might not know, then fill in: don't know.

1,24 How much money do you spend in your household each month (on average)?
Write spending as reported. Young respondents might not know, then fill in: don't know.

1,25 What is the value of your household's current savings/assets?
Write value as reported. Young respondents might not know, then fill in: don't know.

1,26 Who is the primary income-earner in your household?
Select one

- ○ Mother
- ○ Father
- ○ Grandmother
- ○ Grandfather
- ○ Aunt
- ○ Uncle
- ○ Sister(s)
- ○ Brother(s)
- ○ Wife/ husband
- ○ Me
- ○ Other

Specify other.

1,27 How many hours of income-earning activity do you personally do per day on average?
Write how many hours as reported

1,28 Who does most of the cooking, cleaning and childcare in your household?
Do not read the list, tick all that apply

- ☐ Mother
- ☐ Father
- ☐ Grandmother
- ☐ Grandfather
- ☐ Aunt
- ☐ Uncle
- ☐ Sister(s)
- ☐ Brother(s)
- ☐ Wife/ husband
- ☐ Me
- ☐ Other

Specify other.

1,29 How many hours of cooking, cleaning and childcare do you personally do per day on average?
Write how many hours as reported

1,30 Who makes the decisions about spending money in your household?
Do not read the list, only if the respondent provides multiple answers please ask him or her to mention the MAIN DECISION MAKER

- ◯ Mother
- ◯ Father
- ◯ Grandmother
- ◯ Grandfather
- ◯ Aunt
- ◯ Uncle
- ◯ Sister(s)
- ◯ Brother(s)
- ◯ Wife/ husband
- ◯ Me

Issues around SRHR

» Section 2 - Social surroundings and SRHR

2,01 How do you find it to talk to your parents/guardians about sexuality?

- ◯ I find it easy
- ◯ I find it difficult
- ◯ No answer
- ◯ Other

Specify other.

____

2,01z How do you find it to talk to your parents/guardians about marriage ?

- ◯ I find it easy
- ◯ I find it difficult
- ◯ No answer
- ◯ Other

Specify other.

____

Have you ever discussed with friends and/or family about:
Inde| Ai | SD = Sindidziwa | PY = Palibe yankho

	Yes	No	Don't know	No answer
**2,02 Marriage**	◯	◯	◯	◯
**2,03 Dating and relationships**	◯	◯	◯	◯
**2,04 How to prevent pregnancies**	◯	◯	◯	◯
**2,05 What it means to be circumcised**	◯	◯	◯	◯
**2,06 What it means to be out of school**	◯	◯	◯	◯
**2,07 Your questions about sexuality and sexual health**	◯	◯	◯	◯
**2,08 Your hopes and fears about the future**	◯	◯	◯	◯

2,09 Did you ever receive education about sexuality and sexual health?
Inde| Ai | SD = Sindidziwa | PY = Palibe yankho

- ○ Yes
- ○ No
- ○ Don't know
- ○ No answer

2,10 From whom did you receive education about sexuality and sexual health?

- ☐ Parents
- ☐ Other family members
- ☐ Religious leader
- ☐ Health provider
- ☐ Friends
- ☐ Peer educator/ counsellor
- ☐ Youth club
- ☐ Teacher
- ☐ Traditional leader
- ☐ Media (TV, radio, internet, newspaper/ magazine)
- ☐ Other

Specify other.

____

2,11 Where do you prefer to get information about sexuality, sexual health and prevention of pregnancy?
Do not read the list, tick all that apply

- ☐ Home
- ☐ Church/mosque
- ☐ Health centre
- ☐ Friends
- ☐ School
- ☐ TV
- ☐ Radio
- ☐ Internet
- ☐ Newspaper/ magazine
- ☐ Phone
- ☐ Youth club
- ☐ No answer
- ☐ Other

Specify other.

___

2,12 In an average week, which sources of media do you use?
Do not read the list, tick all that apply

- [ ] Television
- [ ] Internet
- [ ] Radio
- [ ] Newspaper
- [ ] Magazine
- [ ] No answer
- [ ] Other

Specify other.

____

2,13 Who in your opinion are important people to promote sexual and reproductive health and rights?
Do not read the list, tick all that apply

- [ ] Teachers
- [ ] Health providers
- [ ] Religious leaders
- [ ] Traditional leaders
- [ ] Youth advocates
- [ ] Peer educators and peer counsellors
- [ ] Don't know
- [ ] Other

Specify other.

2,14 Do you have someone at home with whom you can talk to about your feelings/hopes/worries most of the time?
Inde| Ai | SD = Sindidziwa | PY = Palibe yankho

- Yes
- No
- Don't know
- No answer

2,15 Who is that person?
Please tick only one option

- Mother
- Father
- Sister
- Brother
- Cousin
- Grandfather/mother
- Spouse
- Aunt/uncle
- No answer

» Section 3 - Awareness and access to contraceptive methods and SRH services

3,01 How can a girl prevent pregnancy?
Do not read the list, tick which one is said

- [ ] Abstinence
- [ ] Periodic abstinence
- [ ] Using condoms
- [ ] Birth control pill
- [ ] Injectables
- [ ] Using IUD
- [ ] Use morning after pill/emergency pill
- [ ] Using withdrawal
- [ ] Observing safe days
- [ ] Don't know
- [ ] No answer
- [ ] Other

Specify other.

____

Please indicate whether you agree or disagree with the following statements:
0= Nditsutsa| 1= Ndibvomekezana nazo | SD = Sindidziwa | PY = Palibe yankho

	0	1	DK	NA
**3,02 It is not appropriate for a girl to propose to use a condom**	○	○	○	○
**3,03 It is easy for a boy to propose to use a condom**	○	○	○	○
**3,04 I feel confident that I can insist on condom use every time I have sex**	○	○	○	○
**3,05 It is difficult to access contraceptives for young people**	○	○	○	○
**3,06 It is easy to access contraceptives as a married young person**	○	○	○	○
**3,07 I can decide for myself whom to date and go out with**	○	○	○	○
**3,08 My parents or relative decide my future partner**	○	○	○	○

3,09 Did you ever utilize any of the following services?
Read all services and select the ones used

- ☐ Ante natal & postnatal services
- ☐ Family planning services
- ☐ VCT
- ☐ PMTCT
- ☐ Abortion
- ☐ Post abortion care
- ☐ Life skills and sexuality counselling
- ☐ Child protection services
- ☐ Hotlines

☐ None of these services

☐ Other

Specify other.

____

9,09z1 Have you ever used any contraceptive methods?

◯ Yes

◯ No

9,09z2 Do you currently use any family planning methods?

◯ Yes

◯ No

» Section 4 - Worries, aspirations and entitlements

The following aspects concern the rights of young people especially girls
Ndimada nkhawa ndi... . 0= Nditsutsa| 1= Ndibvomekezana nazo | SD = Sindidziwa | PY = Palibe yankho

	0	1	DK	NA
**4,01 To become/make (someone) pregnant early**	◯	◯	◯	◯
**4,02 To become a bride/groom early**	◯	◯	◯	◯
**4,03 To not finish school**	◯	◯	◯	◯
**4,04 To be worth a bride price only**	◯	◯	◯	◯
**4,04z To be worth a dowry only**	◯	◯	◯	◯
**4,05 To not decide for myself who to date**	◯	◯	◯	◯
**4,06 To be denied access to contraceptives**	◯	◯	◯	◯

4,07 What do you think most about in your life?
Do not read the list, tick what is said

- ☐ Education/completing studies
- ☐ Getting a job/ working/ prospering
- ☐ My health/future
- ☐ Obtaining/taking medication
- ☐ HIV
- ☐ Having spouse/family/children
- ☐ No answer
- ☐ Other

Specify other.

____

4,08 If a girl falls pregnant here, whom can she turn to?
Do not read the list, tick what is said

- ☐ Family members/relatives
- ☐ Health service provider
- ☐ Teachers
- ☐ Religious leaders
- ☐ Youth advocates
- ☐ Partner/boy/girlfriend/friends
- ☐ Other professional staff
- ☐ No answer

☐ Other

Specify other.

____

Section 5 - Gender equality

To what extent do you think or do the following statements:
0= Olo pan'gono pekha | 1= Pan'gono | 2 = Kothelatu | SD = Sindidziwa

	0	1	2	DK
**5,01 I feel confident discussing gender equality and girls' rights with girls my age.**	◯	◯	◯	◯
**5,02 I feel confident discussing gender equality and girls' rights with boys my age**	◯	◯	◯	◯
**5,03 I feel confident discussing gender equality and girls' rights with adult women**	◯	◯	◯	◯
**5,04 I feel confident discussing gender equality and girls' rights with adult men**	◯	◯	◯	◯

Child Marriage, teenage pregnancy and female genital mutilation/ cutting

» Section 6 - Marriage

6,01 What is your marital status?

◯ Single (unmarried)

◯ Single (unmarried) but with boy- or girlfriend

◯ Single (unmarried) but with multiple relationships

◯ Cohabiting

◯ Monogamous married

◯ Polygamous married

◯ Divorced

◯ Widowed

- [ ] Separated

6,02 What are the most common ways of marriage in this community?
Read the list, tick all that apply

- [ ] Formal through register
- [ ] Informal, agreement between families
- [ ] Register with community head
- [ ] Religious marriage - in the mosque
- [ ] Religious marriage - in church
- [ ] Paying of dowry (by the family of the girl)
- [ ] Paying of bride price (by the family of the boy)
- [ ] Other

Specify other.

____

» Section 7 - After marriage

7,01 At what age did you (first) get married?

____

7,02 When exactly did you get married (month and year)?

mm/dd/yyyy

7,03 What age was your partner at that time?

____

7,04 Looking back do you feel that this was the right time for you to have been married?
Inde| Ai | SD = Sindidziwa | PY = Palibe yankho

- Yes
- No
- Don't know
- No answer

7,05 Was it your choice to get married at this time?
Inde| Ai | SD = Sindidziwa | PY = Palibe yankho

- Yes
- No
- Don't know
- No answer

7,06 Did you feel pressured into marriage by any person or your family?
Inde| Ai | SD = Sindidziwa | PY = Palibe yankho

- Yes
- No
- Don't know
- No answer

7,07 Did you feel any social pressure/ pressure from society to get married?
Inde| Ai | SD = Sindidziwa | PY = Palibe yankho

- Yes
- No
- Don't know
- No answer

7,08 Does/ did your partner ever physically hurt or hit you?
Read the list, tick one

- ◯ All the time
- ◯ Frequently
- ◯ Sometimes
- ◯ Rarely
- ◯ Never
- ◯ I don't wish to share this

7,09 How often do you experience sexual harassment / 'eve teasing'?
Read the list, tick one

- ◯ Everyday
- ◯ Once or twice a week
- ◯ Once or twice a month
- ◯ Less than once a month
- ◯ Never
- ◯ I don't wish to share this

7,10 Was your (first) marriage registered?
Read the list, tick all that apply

- ☐ No registration
- ☐ Religious registration or rituals

- [ ] Government (legal) registration

7,11 Do you have a copy of your marriage certificate now?

Inde| Ai | SD = Sindidziwa | PY = Palibe yankho

- ○ Yes
- ○ No
- ○ Don't know
- ○ No answer

7,12 Of all marriages in your community, what is the proportion/ percentage of marriages where one person is under 18 years?

Read the list, tick one

- ○ All
- ○ Most
- ○ About half
- ○ A few
- ○ None
- ○ Don't know

7,13 Of all marriages in your community what is the proportion/ percentage that happens without the willingness of the bride?

Read the list, tick one

- ○ All
- ○ Most
- ○ About half
- ○ A few
- ○ None
- ○ Don't know

7,14 Of all marriages in your community what is the proportion/ percentage that happens without the willingness of the groom?

Read the list, tick one

- All
- Most
- About half
- A few
- None
- Don't know

7,15 Of all marriages in your community what is the proportion/ percentage that involves an agreement concerning the exchange of money, goods or other benefits (e.g. dowry)?

Read the list, tick one

- All
- Most
- About half
- A few
- None
- Don't know

» Section 8 - Single

8,01 (If any) At what age do you want to be married?

- Age
- Never want to marry
- No defined age

8,02 Age

8,02 Age

____

8,03 Who would you turn to in case someone pressurizes you into getting married?

- [ ] Parents
- [ ] Siblings
- [ ] Uncle
- [ ] Aunt
- [ ] Grandparents
- [ ] Teacher
- [ ] Traditional leader
- [ ] Religious leader
- [ ] Youth group
- [ ] Friend
- [ ] Other

Specify other.

____

8,04 Of all marriages in your community, what is the proportion/ percentage of marriages under 18 years?
Read the list, tick one

- ○ All
- ○ Most
- ○ About half
- ○ A few
- ○ None

- Don't know

8,05 Of all marriages in your community what is the proportion/ percentage that happens without the willingness of the bride?

Read the list, tick one

- All
- Most
- About half
- A few
- None
- Don't know

8,06 Of all marriages in your community what is the proportion/ percentage that happens without the willingness of the groom?

Read the list, tick one

- All
- Most
- About half
- A few
- None
- Don't know

8,07 Of all marriages in your community what is the proportion/ percentage that involves an agreement concerning the exchange of money, goods or other benefits (e.g. dowry)?

Read the list, tick one

- All
- Most
- About half
- A few
- None
- Don't know

8,08 How often do you experience sexual harassment / 'eve teasing'?

Read the list, tick one

- Everyday
- Once or twice a week
- Once or twice a month
- Less than once a month
- Never
- I don't wish to share this

» Section 9 - Teenage pregnancy

9,01 Do you have or ever had any children?

- Yes
- No

9,02 What is the birth date of your first born (month and year)?

mm/dd/yyyy

9,03 How many pregnancies have you had?

____

9,04 How many of your children live with you?

____

9,05 What was your age at your first pregnancy?

____

9,06 At what age did you become a father?

____

9,07 Did you want to become a parent at this time?
Inde| Ai | SD = Sindidziwa | PY = Palibe yankho

- ◯ Yes
- ◯ No
- ◯ Don't know
- ◯ No answer

9,08 How many girls do you know that have had children before the age of 18?

____

9,09 What method of contraception do you use?

Do not read the list, tick what is said

- [ ] None
- [ ] Natural family planning
- [ ] Male condom
- [ ] Female condom
- [ ] Diaphragm
- [ ] Contraceptive pill
- [ ] Injections
- [ ] Implant
- [ ] IUD
- [ ] Sterilization
- [ ] Don't know
- [ ] Other

Specify other.

____

9,10 What types of contraception are available in the area where you live?

Do not read the list, tick what is said

- [ ] None
- [ ] Natural family planning
- [ ] Male condom
- [ ] Female condom
- [ ] Diaphragm
- [ ] Contraceptive pill
- [ ] Injections
- [ ] Implant
- [ ] IUD
- [ ] Sterilization
- [ ] Don't know
- [ ] Other

Specify other.

____

9,11 When can a person access a 'modern' form of contraception? (Any form of contraception except natural family planning)

Read the list, tick which one is said

- ◯ Always
- ◯ Never
- ◯ Only once married
- ◯ Only after having children
- ◯ At the age of puberty
- ◯ At a specific age

9,12 Specific age

____

» Section 10 - (Child) marriage: norms, perceptions and attitudes

10,01 In your opinion at what age does a GIRL

become adult?

____

10,02 and a BOY?

____

10,03 What is the average age that WOMEN live to in your community? (Life expectancy)

____

10,04 and MEN?

____

10,05 In your opinion what is the ideal age for GIRLS to get married?

____

[illegible]

10,06 and for BOYS?

____

10,07 In your opinion what is too old for GIRLS to get married?

____

10,08 and for BOYS?

____

10,09 In your opinion what is too young for GIRLS to get married?

____

10,10 and for BOYS?

____

What is the level of your agreement with the following statements?

0= Ndikanitsitsa| 1= Ndikana | 2= Ndikanako pang'ono | 3= Zilipakatimpakati | 4= Ndizoonako | 5= Ndizoona | 6= Ndizoona kwambiri | SD= Sindidziwa

	0	1	2	3	4	5	6	DK
**10,11 A GIRL is ready for marriage once she starts menstruating**	○	○	○	○	○	○	○	○
**10,12 There are advantages to marriage under 18 years for GIRLS**	○	○	○	○	○	○	○	○
**10,13 and for BOYS**	○	○	○	○	○	○	○	○
**10,14 There are DIS-advantages to marriage under 18 years for GIRLS**	○	○	○	○	○	○	○	○
**10,15 and for BOYS**	○	○	○	○	○	○	○	○
**10,16 Marrying GIRLS below the age of 18 is the tradition/ culture in this society**	○	○	○	○	○	○	○	○
**10,17 and BOYS**	○	○	○	○	○	○	○	○

What is the level of your agreement with the following statements?

0= Ndikanitsitsa| 1= Ndikana | 2= Ndikanako pang'ono | 3= Zilipakatimpakati | 4= Ndizoonako | 5= Ndizoona | 6= Ndizoona kwambiri | SD= Sindidziwa

	0	1	2	3	4	5	6	DK
**10,18 Marrying GIRLS at a young age is part of our religious practices**	○	○	○	○	○	○	○	○
**10,19 and BOYS**	○	○	○	○	○	○	○	○
**10,20 Marrying GIRLS young can help protect family honour/ reputation**	○	○	○	○	○	○	○	○
**10,21 and BOYS**	○	○	○	○	○	○	○	○
**10,22 Marriage of GIRLS under 18 years may happen because of pregnancy in your community**	○	○	○	○	○	○	○	○
**10,23 and of BOYS**	○	○	○	○	○	○	○	○

What is the level of your agreement with the following statements?

0= Ndikanitsitsa| 1= Ndikana | 2= Ndikanako pang'ono | 3= Zilipakatimpakati | 4= Ndizoonako | 5= Ndizoona | 6= Ndizoona kwambiri | SD= Sindidziwa

	0	1	2	3	4	5	6	DK
**10,24 Marriage of GIRLS under 18 years may happen to resolve family disputes**	○	○	○	○	○	○	○	○
**10,25 and of BOYS**	○	○	○	○	○	○	○	○
**10,26 GIRLS who give birth between 15-18 years are more likely to have a healthy pregnancy/ baby (compared to girls over 18), because their bodies are young and strong**	○	○	○	○	○	○	○	○
**10,27 Marriage of GIRLS under 18 years mostly happens because of love**	○	○	○	○	○	○	○	○
**10,28 and of BOYS**	○	○	○	○	○	○	○	○

What is the level of your agreement with the following statements?

0= Ndikanitsitsa| 1= Ndikana | 2= Ndikanako pang'ono | 3= Zilipakatimpakati | 4= Ndizoonako | 5= Ndizoona | 6= Ndizoona kwambiri | SD= Sindidziwa

	0	1	2	3	4	5	6	DK
**10,29 Marriage of GIRLS under 18 years mostly happens because there is a lack of education and job opportunities**	○	○	○	○	○	○	○	○
**10,30 and of BOYS**	○	○	○	○	○	○	○	○
**10,31 Marrying GIRLS can help resolve financial problems in the family**	○	○	○	○	○	○	○	○
**10,32 and BOYS**	○	○	○	○	○	○	○	○
**10,33 Marrying GIRLS at a young age can help provide them security**	○	○	○	○	○	○	○	○
**10,34 and BOYS**	○	○	○	○	○	○	○	○

What is the level of your agreement with the following statements?

0= Ndikanitsitsa| 1= Ndikana | 2= Ndikanako pang'ono | 3= Zilipakatimpakati | 4= Ndizoonako | 5= Ndizoona | 6= Ndizoona kwambiri | SD= Sindidziwa

	0	1	2	3	4	5	6	DK
**10,35 Younger BRIDES often require a lower dowry than older brides**	○	○	○	○	○	○	○	○
**10,36 Marriage of GIRLS under 18 years sometimes happens for financial reasons**	○	○	○	○	○	○	○	○
**10,37 and of BOYS**	○	○	○	○	○	○	○	○
**10,38 Marrying GIRLS young can help prevent sexual violence, assault and harassment**	○	○	○	○	○	○	○	○
**10,39 and BOYS**	○	○	○	○	○	○	○	○
**10,40 GIRLS who become pregnant under 18 years are more likely to have health problems, and/or miscarry (compared to girls over 18 years)**	○	○	○	○	○	○	○	○

What is the level of your agreement with the following statements?

0= Ndikanitsitsa| 1= Ndikana | 2= Ndikanako pang'ono | 3= Zilipakatimpakati | 4= Ndizoonako | 5= Ndizoona | 6= Ndizoona kwambiri | SD= Sindidziwa

	0	1	2	3	4	5	6	DK
**10,41 Marriage under 18 years is likely to have a negative impact on a GIRLS education**								
**10,42 and on BOYS**								
**10,43 Marrying a GIRL young is preferable because younger brides are more obedient and respectful of their husbands**								
**10,44 Even if a GIRL does not want to be married she should honour the decisions/ wishes of her family.**								
**10,45 and if a BOY**								
**10,46 It is sometimes ok to physically beat or punish a GIRL if she dishonours her family**								
**10,46z It is sometimes ok to physically beat or punish a BOY if he dishonours his family**								

What is the level of your agreement with the following statements?

0= Ndikanitsitsa| 1= Ndikana | 2= Ndikanako pang'ono | 3= Zilipakatimpakati | 4= Ndizoonako | 5= Ndizoona | 6= Ndizoona kwambiri | SD= Sindidziwa

	0	1	2	3	4	5	6	DK
**10,47 A GIRL should be allowed to choose for herself whom to marry**								
**10,48 and a BOY**								
**10,49 A GIRL should never be forced or compelled into marriage**								
**10,50 and a BOY**								
**10,51 A WIFE should be subservient to her husband**								
**10,52 MEN should be the heads of their household**								
**10,53 A MAN should only marry one wife**								

10,54 GIRLS who are married under 18 years are often married to men who already have a wife/wives ○ ○ ○ ○ ○ ○ ○ ○

» Section 11- Marriage law

11,01 Do you know - is there a legal minimum age for marriage according to statutory law?
Inde| Ai | SD = Sindidziwa | PY = Palibe yankho

- ○ Yes
- ○ No
- ○ Don't know
- ○ No answer

11,02 What is the minimum age for marriage according to statutory law for girls?

____

11,03 What is the minimum age for marriage according to statutory law for boys?

____

11,04 Do children ever get married under this legal age?
Read the list, tick which one is said

- ○ All the time
- ○ Frequently
- ○ Sometimes
- ○ Rarely
- ○ Never
- ○ I don't wish to share this

11,05 When children are married or about to be married under the legal age, does anyone intervene?
Inde| Ai | SD = Sindidziwa | PY = Palibe yankho

- ○ Yes
- ○ No
- ○ Don't know

No answer

11,06 Who usually intervenes?
Do not read the list, tick all that apply

- Police
- Law enforcement agents
- Community leaders (e.g. ‘influentials’)
- NGO staff
- Other

Specify other.

11,07 In what proportion/ percentage of cases do they intervene?
Read the list, tick which one is said

- All the time
- Frequently
- Sometimes
- Rarely
- Never
- I don't wish to share this

11,08 Is there/ what is the minimum age for marriage according to the law of your religion? For girls
Read the list, tick which one is said

- No minimum age
- Age of puberty
- Specific age
- Don't know

11,09 Specific age

____

11,10 Is there/ what is the minimum age for marriage according to the law of your religion? For boys
Read the list, tick which one is said

- ◯ No minimum age
- ◯ Age of puberty
- ◯ Specific age
- ◯ Don't know

11,11 Specific age

____

11,11z1 Is there/ what is the minimum age for marriage according to the law of your tribe? For girls
Read the list, tick which one is said

- ◯ No minimum age
- ◯ Age of puberty
- ◯ Specific age
- ◯ Don't know

11,11z2 Specific age

____

11,11z3 Is there/ what is the minimum age for marriage according to the law of your tribe? For boys
Read the list, tick which one is said

- ◯ No minimum age
- ◯ Age of puberty
- ◯ Specific age
- ◯ Don't know

11,11z4 Specific age

11,12 Is there anything else that you would like to share at this time?

» Section 12 - Opinions about child marriage

What do you think about child marriage?
Inde| Ai | SD = Sindidziwa | PY = Palibe yankho

	Yes	No	Don't know	No answer
**12,01 The benefits are for the family not for the child**	○	○	○	○
**12,02 It is a solution if a girl gets pregnant**	○	○	○	○
**12,04 It often occurs after a teenage pregnancy**	○	○	○	○
**12,05 I know many girls been married against their wish**	○	○	○	○
**12,06 I know how to prevent pregnancies**	○	○	○	○
**12,07 When married, a girl drops out of school**	○	○	○	○
**12,08 When drop out of school, girls and boys have no access to information**	○	○	○	○
**12,09 Economic empowerment is a solution against CM**	○	○	○	○
**12,10 Boys and men should act together in tackling CM**	○	○	○	○

THANK YOU FOR YOUR TIME

Any questions?

Any questions?

14,01 Do you have any questions

Interviewer's comments

15,01 Interviewer's comments

---

000 Please register the location

latitude (x.y °) longitude (x.y °) altitude (m) accuracy (m)

ODKSurvey_Zambia_FINAL

NDIME YA 0 - Mau oyambilira komanso nkhani zokhudza umoyo wa za chimuna ndi ukazi

0,01 Please register the location

latitude (x.y °) longitude (x.y °) altitude (m) accuracy (m)

0,02 In which district are you located?

required

- (●) Chadiza
- ( ) Petauke
- ( ) Katete

0,02z1 In which ward are you located?

- ( ) Chanjowe
- ( ) Chilenga
- ( ) Kabvumo
- ( ) Kandabwako
- ( ) Kapachi
- ( ) Mangwe
- ( ) Manje
- ( ) Naviluri
- ( ) Nkhumba
- ( ) Nsadzu
- ( ) Taferansoni

0,02z2 In which ward are you located?

- ◯ Chalimanyama
- ◯ Kovyane
- ◯ Mateyo Mzeka
- ◯ Mawanda
- ◯ Mbala
- ◯ Msumbazi
- ◯ Nsimbo
- ◯ Nyakawise
- ◯ Nyika
- ◯ Ongolwe
- ◯ Singozi
- ◯ Ukwimi

0,02z3 In which ward are you located?

- ◯ Chavuka
- ◯ Chimtende
- ◯ Chitawe
- ◯ Chiwuyu
- ◯ Dole
- ◯ Kadula
- ◯ Kafumbwe
- ◯ Kapangulula
- ◯ Kapoche
- ◯ Katiula
- ◯ Mkaika
- ◯ Mngo'mba
- ◯ Mnyamazi
- ◯ Mphangwe
- ◯ Mwandafisi
- ◯ Nyamasonkho
- ◯ Sinda
- ◯ Vulamkoko

0,02z4 In which CSA are you located?

0,02z5 In which SEA are you located?

0,03 Ask for consent to take part in the interview

Please explain the objective and consequences of participation to this survey to the respondent. If minor, has the main caregiver/ guardian of the family provided consent for the interview?

- ◯ Chivomelezo
- ◯ Ukana

0,04 Why did the person refuse to participate?

0,05 Interviewers name

- ◯ Candy Kunda
- ◯ Cynthia Simakoloyi
- ◯ Glenda Chileshe
- ◯ Grace Nalikando
- ◯ Helen Mtonga
- ◯ Isaac Jerry
- ◯ Mable Maboshe
- ◯ Mabvuto Tembo
- ◯ Musonda Mwape
- ◯ Nguli Zulu
- ◯ Pungashi Manda
- ◯ Raquel Kazembe
- ◯ Sarah Kamanga
- ◯ Sarah Mbobola
- ◯ Sheila Khonje
- ◯ Yvone Chileshe

NDIME YA 1 - Mbiri ya munthu amene akufunsidwa mafunso

1,01 Ndi mwamuna kapena mkazi

- ◯ Mkazi

◯ Mwamuna

1,02 Kodi muli ndi zaka zingati tsopano?

◯ Zaka

◯ Sindidziwa

◯ Palibe yankho

1,03 Zaka

____

1,04 Kodi muli ndi chinkhope kapena chipepala copatsidwa pobadwa?
Inde| Ai | SD = Sindidziwa | PY = Palibe yankho

◯ Inde

◯ Ai

◯ SD

◯ PY

1,05 Kodi muli ku mpingo kapena ku chipembedzo cina ciliconse?
Inde| Ai | SD = Sindidziwa | PY = Palibe yankho

◯ Inde

◯ Ai

◯ SD

◯ PY

1,06 Ndi chipembedzo citi comwe mutengako mbali?

- African methodist
- UCZ
- 7th day adventist
- Reformed church
- Baptist
- New apostolic
- CMML
- Salvation army
- Jehovah’s witness (watchtower)
- Pentecostal
- Presbyterian
- Anglican
- Catholic
- Muslim
- Hindu
- Other

Specify other.

1,07 Ndimwe atundu bwanji?

- ◯ Bemba
- ◯ Lozi
- ◯ Tonga
- ◯ Kaonde
- ◯ Lunda
- ◯ Luvale
- ◯ Other

Specify other.

____

1,08 Mukhala ndi ndani?

You may mark more than 1 option

- [ ] Amai
- [ ] Atate
- [ ] Apongozi anga akazi
- [ ] Apongozi anga amuna
- [ ] Ambuya akazi
- [ ] Ambuya amuna
- [ ] Alongo abambo wanga acikazi/alongo aa mai wanga acikazi
- [ ] Amalume
- [ ] Mlongo kapena alongosi anga acikazi
- [ ] M'longo wanga kapena alongosi anga acimuna
- [ ] Ndi mkazi wanga/ndi mwamuna wanga
- [ ] Palibe amene ndikhala naye
- [ ] Other

Specify other.

____

1,09 Kukula kwa banja

This should be the total number of people who eat together/ live together as a family unit. Write down "1" if respondent lives alone.

____

1,10 Kodi munafika potani mkhani ya maphunzilo?

Please mark only one option

- ◯ Palibe maphunziro
- ◯ Sukulu yapansi ya pulayimale
- ◯ Sukulu yapamwamba ya pulayimale
- ◯ Sukulu ya pansi ya sekondale
- ◯ Sukulu yapamwamba ya sekondale
- ◯ Maphunziro ya zabvopanga panga mpnga ukalipentala
- ◯ Maphunziro yapamwamba yakuya

1,10z Ndi sukulu yotani yomwe munapitako?

- ◯ Sukulu ya Boma
- ◯ Sukulu yomwe siyili ya Boma
- ◯ Other

Specify other.

____

1,11 Kodi munatsiliza zaka zingati muli pasukulu?

Number of years starting from primary school, do not count repeated years

____

1,12 Kodi ndimaphunziro otani omwe mukucita kwatsopano lino?

Please mark only one option

- ◯ Sukulu yapansi ya pulayimale
- ◯ Sukulu yapamwamba ya pulayimale
- ◯ Sukulu ya pansi ya sekondale
- ◯ Sukulu yapamwamba ya sekondale
- ◯ Maphunziro yapamwamba
- ◯ Maphunziro yapamwamba yakuya
- ◯ Anasiliza
- ◯ Other

Specify other.

____

1,13 Kodi munalekeza sukulu ndipo munalekeza pazifukwa zotani?

Do not read the list, tick all that apply

- ☐ Ai
- ☐ Kusowekela kwa ndalama/zofunikira zina za kusukulu
- ☐ Nchito zapanyumba/zapabanja
- ☐ Kudwala
- ☐ Makolo anamwalira ndikali wamng'ono
- ☐ Ndinatenga pathupi/ndinakhala ndi mwana
- ☐ Sukulu ili kutali/kulibe sukulu
- ☐ Ndilibe nthawi
- ☐ Sindinakonde sukulu
- ☐ Ndinakwatiliwa
- ☐ Ndinaimitsidwa/kucotsedwa
- ☐ Ndinkaopa kusulidwa/kusalidwa
- ☐ Sindikumbukira
- ☐ Palibe yankho
- ☐ Other

Specify other.

____

1,14 Kodi amai ako anafika potani mnkhani yamaphunziro?

- Alibe maphunziro
- Sukulu yapansi ya pulayimale
- Sukulu yapamwamba ya pulayimale
- Sukulu ya pansi ya sekondale
- Sukulu yapamwamba ya sekondale
- Maphunziro ya zabvopangapanga monga ukalipentela
- Maphunziro yapamwamba yakuya
- Sindidziwa

1,15 Kodi amai ako anatsliza zaka zingati zasukulu?

Number of years starting from primary school. IF RESPONDENT DOES NOT KNOW TYPE 'don't know'

1,16 Kodi atate ako anafika potani mnkhani yamaphunziro?

- Alibe maphunziro
- Sukulu yapansi ya pulayimale
- Sukulu yapamwamba ya pulayimale
- Sukulu ya pansi ya sekondale
- Sukulu yapamwamba ya sekondale
- Maphunziro ya zabvopangapanga monga ukalipentela
- Maphunziro yapamwamba yakuya
- Sindidziwa

1,17 Kodi atate ako anatsliza zaka zingati zasukulu?

Number of years starting from primary school. IF RESPONDENT DOES NOT KNOW TYPE 'don't know'

1,18 Kodi munalandilako malipiro (ndalama)ali onse mu minyezi isanu ndi umodzi yomwe yapita kumbuyo uku?

Inde| Ai | SD = Sindidziwa | PY = Palibe yankho

- ○ Inde
- ○ Ai
- ○ SD
- ○ PY

1,19 Ngati munalandilako malipiro (ndalama) ali onse paminyezi isanu ndi umodzi yomwe yapita kumbuyo uku, kodi malipiliro amenewo anacokera kuti?

You may mark more than 1 option

- ☐ Amai
- ☐ Atate
- ☐ Alongo a bamboo wanga acikazi/alongo a mai wanga acikazi (Aunt)
- ☐ Amalume
- ☐ Mlongo wacikazi
- ☐ Mlongo wacimuna
- ☐ Apongozi akazi/amuna
- ☐ Ku panchito yomwe siyamuyaya (nchito yosakhalitsapo)
- ☐ Panchito yolembedwa yamuyaya
- ☐ Kumalonda yanga
- ☐ Thandizo ya ndalama yocokela ku Boma
- ☐ Other

Specify other.

1,20 Mumagwira nchito yotani?

Do not read the list, tick all that apply

- ☐ Sali pa nchito
- ☐ Sali pa nchito yolipilidwa (mwacitsanzo; makzi wapanyumba)
- ☐ Mlimi wa mng'ono
- ☐ Wamalonda wa mng'ono
- ☐ Wogwira nchito yolipilidwa patsiku
- ☐ Wogwira nchito ya nthawi yopimidwa monga zaka zibili olo zitatu
- ☐ Wodzilemba yekha nchito
- ☐ Wogwira nchito yomwe maliro alingana ndi nchito imene apatsidwa (ya temparare)
- ☐ Wogwira nchito yamuyaya amene ali pamalipiro ya pamwezi

1,21 Kodi ndi nchito yotani yomwe mugwira?

Do not read the list, tick all that apply

- [ ] Wa zaulimi
- [ ] Mlimi wa ziweto
- [ ] Wocita malonda mwapatali-patali
- [ ] Mwiniwace wanchito zamalondo ang’ono-ang’ono
- [ ] Mthandizi wa panyumba. (Munthu wacikazi amene agwira nchito panyumba pawina/Mnyamata amene asewenza panyumba ya wina monga nchito ya mdima)
- [ ] Wapanchito yomwe siyamuyaya (lebara)
- [ ] Wazomanga-manga
- [ ] Nchito za zopangapanga
- [ ] Nchito zolukaluka
- [ ] Nchito zachipiku ndi kugulitsa cimodzi-cimodzi
- [ ] Nyumba zamalonda zogonamo ndi zogulitsamo zakudya
- [ ] Nchito za mtengatenga ndi mtokoma
- [ ] Nchito zogulitsa manyumba ndi kuika manyumba pa lende
- [ ] Kulandira ndalama kucokera kunja kwa dziko
- [ ] Mnyanchityo wa Boma
- [ ] Wosewenza ku mabungwe odziimila pa okha anchito zothandiza anthu
- [ ] Other

Specify other.

1,22 Kodi ni zinthu zotani zomwe zibweletsa ndalama panyumba?
Do not read the list, tick all that apply

- [ ] Wa zaulimi
- [ ] Mlimi wa ziweto
- [ ] Wocita malonda mwapatali-patali
- [ ] Mwiniwace wanchito zamalondo ang'ono-ang'ono
- [ ] Mthandizi wa panyumba. (Munthu wacikazi amene agwira nchito panyumba pawina/Mnyamata amene asewenza panyumba ya wina monga nchito ya mdima)
- [ ] Wapanchito yomwe siyamuyaya (lebara)
- [ ] Wazomanga-manga
- [ ] Nchito za zopangapanga
- [ ] Nchito zolukaluka
- [ ] Nchito zachipiku ndi kugulitsa cimodzi-cimodzi
- [ ] Nyumba zamalonda zogonamo ndi zogulitsamo zakudya
- [ ] Nchito za mtengatenga ndi mtokoma
- [ ] Nchito zogulitsa manyumba ndi kuika manyumba pa lende
- [ ] Kulandira ndalama kucokera kunja kwa dziko
- [ ] Mnyanchityo wa Boma

☐ Wosewenza ku mabungwe odziimila pa okha anchito zothandiza anthu

☐ Other

Specify other.

____

1,23 Kodi ndi ndalama zokwanira ngati zingati zomwe banja lanu limapeza pamwezi uli onse?
Write income as reported. Young respondents might not know, then fill in: don’t know.

____

1,24 Kodi ndi ndalama zokwanira ngati zingati zomwe banja lanu limagwilitsa nchito panyumba pa mwezi umodzi?
Write spending as reported. Young respondents might not know, then fill in: don’t know.

____

1,25 Ndi ndalama zokwanira ngati zingati zomwe banja lanu likusunga pa nthawi ino?
Write value as reported. Young respondents might not know, then fill in: don’t know.

____

1,26 Kodi mumadalira ndani m’banja lanu lino kupeza ndalama pano panyumba?
Select one

◯ Amai

◯ Atate

◯ Ambuya

◯ Agogo

◯ Aunt

◯ Amalume

◯ Mlongo

◯ M’bale

◯ Akazi anga/ amuna anga

◯ Ine mwini

- ◯ Other

Specify other.

____

1,27 Kodi ndi maola angati omwe inuyo mumakhala mugwira zinchito zobweletsa chuma (ndalama) pano panyumba patsiku ili yonse?
Write how many hours as reported

____

1,28 Kodi ndani kawiri-kawiri amene amaphika, kusamalira pa nyumba ndi kusamalira ana pa nyumba panu?
Do not read the list, tick all that apply

- ☐ Amai
- ☐ Atate
- ☐ Ambuya
- ☐ Agogo
- ☐ Aunt
- ☐ Amalume
- ☐ Mlongo
- ☐ M'bale
- ☐ Akazi anga/ amuna anga
- ☐ Ine mwini
- ☐ Other

Specify other.

____

1,29 Kodi inuyo mumatenga maola angati kuphika, kusamalira pa nyumba ndi kusamalira ana patsiku ili yonse?
Write how many hours as reported

1,30 Kodi pano panyumba panu, ndani amene amapanga ciganizo kapena chigamulo mukagwilitsidwe nchito kandalama?

Do not read the list, only if the respondent provides multiple answers please ask him or her to mention the MAIN DECISION MAKER

- Amai
- Atate
- Ambuya
- Agogo
- Aunt
- Amalume
- Mlongo
- M'bale
- Akazi anga/ amuna anga
- Ine mwini

Khani zokhuza SRHR

» NDIME YA 2 - Mkhalidwe ndi mauphungu pa umoyo wa za cimuna ndi cikazi

2,01 Kodi mumacipeza cinthu cotani chokambilana ndi makolo anu/omusungani pankhani zo khuza za amuna ndi akazi?

- Ndimacipeza cinthu cha pafupi ndiponso cosabvuta konse
- Ndimacipeza cinthu cobvuta
- Palibe yankho
- Other

Other

Specify other.

2,01z Kodi mumacipeza cinthu cotani chokambilana ndi makolo anu/omusungani pa nkhani ya chikwati?

- Ndimacipeza cinthu cha pafupi ndiponso cosabvuta konse
- Ndimacipeza cinthu cobvuta
- Palibe yankho
- Other

Specify other.

Kodi munalankhuzanapo kale ndi abwenzi anu kapenanso wina wace aliyense wa m'banja lanu pa nkhani ya:
Inde| Ai | SD = Sindidziwa | PY = Palibe yankho

	Inde	Ai	SD	PY
**2,02 Chikwati**	○	○	○	○
**2,03 Uyenda pamodzi ndi chibwenzi pakati pa mwamuna ndi mkazi**	○	○	○	○
**2,04 Momwe mungapewele kutengera pathupi/pakati/mimba**	○	○	○	○
**2,05 Comwe citanthauza kucita mdulidwe**	○	○	○	○
**2,06 Comwe citanthauza kukhala cosapita ku sukulu**	○	○	○	○
**2,07 Mafunso anu pankhani yokhuza za amuna ndi akazi ndi momwe ungadzisungile ndi umoyo wabwino pa nkhani yomwei**	○	○	○	○
**2,08 Ziyembekezero zanu komanso mantha yanu pa za umoyo wanu mtsogolo**	○	○	○	○

2,09 Kodi munalandilako maphunziro ena ali onse pankhani zo khuza za amuna ndi akazi ndi momwe mungadzisungile ndi umoyo wa bwino pali nkhani yomwe iyi?

Inde| Ai | SD = Sindidziwa | PY = Palibe yankho

- Inde
- Ai
- SD
- PY

2,10 Kodi maphunziro amenewa okhuza nkhani za umuna ndi ukazi ndi modzisungila ndi umoyo wa bwino pa nkhani yomwe iyi, anapelekedwa ndi yani kwa inu?

- Panyumba pathu (kucokera kumakolo, abale anga omwe tikhala onse panyumba)
- Ena ace a m'banja lathu
- Akulu a mpingo
- Wazinchito za umoyo
- Anzanga
- Mphunzitsi wanchito za umoyo wa chinyamata
- Ku kalabu ya achinyamata ndi acitsikana
- Mphunzitsi wa kusukulu
- Mafumu amadela a kumidzi
- Panyumba zofalitsa (kanema wa TV, wailesi, maluso amakono ya internet, mapepala ndi mabuku ofalitsa nkhani)
- Other

Specify other.

2,11 Kodi ndi kuti komwe mumakonda kulandilako uthenga pankhani ya za amuna ndi za akazi ndi umoyo okhuza nkhani yomwe iyi, komanso zinjira zomwe mungapelewe kutenga pathupi/pakati/mimba?

Do not read the list, tick all that apply

- [ ] Pa nyumba
- [ ] Ku chalichi/Ku msikitini
- [ ] Ku malo opelekera za umoyo
- [ ] Azanga
- [ ] Ku sukulu
- [ ] Pa kanema woonelera wa TV
- [ ] Pa wailesi
- [ ] Pamaluso amasiku ano a Internet
- [ ] Pa mapepala ofalitsa nkhani ndiponso zolembedwa mu mabuku a magazine
- [ ] Lamia
- [ ] Ku kalabu ya a chinyamata ndi a chitsikana
- [ ] Palibe yankho
- [ ] Other

Specify other.

____

2,12 Kawiri-kawiri msabata imodzi, ndi kuti komwe mumakonda kumvelera?

Do not read the list, tick all that apply

- [ ] Wailesi ya kanema (television)
- [ ] Maluso yamakono ya internet
- [ ] Wailesi
- [ ] Pepala zofalitsa nkhani
- [ ] Magazini
- [ ] Palibe yankho
- [ ] Other

Specify other.

____

2,13 Kulingana ndi maganizo anu, kodi ndani amene ali oyenera utukula ndi kupeleka patsogolo maphunzilo a umoyo wa bwino wokhuza nkhani ya za amuna ndi akazi ndi nkhani ya ubeleki ndiponso kulimbikitsa ma ufulu omwe anthu ali nao pankhani zomwezi?

Do not read the list, tick all that apply

- [ ] Aziphunzitsi
- [ ] A nchito za umoyo
- [ ] Azikulu a mipingo
- [ ] Mafumu a mbali zakumidzi
- [ ] Bolankhulilako ana acicepele (youth advocates)
- [ ] Aphunzitsi a achinyamata komanso athandizi ankhani zokhuza ani acicepele
- [ ] Sindidziwa
- [ ] Other

Specify other.

____

2,14 Kodi pali munthu pa nyumba panu amene mumakamba naye pa nkhani ya momwe inuyo mumamvelera, ziyembekezo zanu komanso pa zomwe zikudetsani nkhawa zambiri?

Inde| Ai | SD = Sindidziwa | PY = Palibe yankho

- ◯ Inde
- ◯ Ai
- ◯ SD
- ◯ PY

2,15 Kodi munthu amene inu mukamba naye ameneyo ndani?

Please tick only one option

- ◯ Amai
- ◯ Atate
- ◯ Kalongosi wacikazi
- ◯ M'bale wacimuna
- ◯ Msuweni
- ◯ Agogo amuna/ambuya akazi
- ◯ Apongozi/ akazi
- ◯ Alomgo a mai olo tate wanga akazi/amalume
- ◯ Palibe yankho

» NDIME YA 3 - Zozdiwika pa cilezi, mapezedwe ya cilezi komanso malo komwe munthu angalandilire mauphungu pankhani ya umoyo wa za umuna ndi ukazi

3,01 Kodi mkazi angapewe bwanji kutenga pathupi/pakati/mimba?

Do not read the list, tick which one is said

- [ ] Kudziletseratu ugona ndi mwamuna
- [ ] Kudziletsa mwakanthawi-kanthawi
- [ ] Kugwilitsa nchito mphira za kondomu
- [ ] Kugwiritsa nchito mapilisi ya chilezi
- [ ] Kugwiritsa nchito mankhwala yachilezi yocita kulasa nsingano
- [ ] Kugwilitsa nchito kachida kolowetsa mu ukazi
- [ ] Mibulu ya mapilisi ya chilezi yakumwa m'mawa mwake utagonana ndi mwamuna
- [ ] Kuchotsa umuna mu ukazi popanda mwamuna popanda kuthilamo mphamvu ya umuna mu ukazi.
- [ ] Kusagonana m'masiku omwe ndiodziwika kuti mkazi atha kutenga pathupi
- [ ] Sindidziwa
- [ ] Palibe yankho
- [ ] Other

Specify other.

____

Chonde fotokozani ngati mubvomerezana kapena simubvomerezana ndi mfundo zomwe zapelekedwa munsimu:
0= Nditsutsa| 1= Ndibvomekezana nazo | SD = Sindidziwa | PY = Palibe yankho

	0	1	SD	PY
**3,02 Sakwabwino kapena sicoyenera kuti munthu wamkazi kuuza mwamuna kuti awilio agwilitse nchito mphira za kondomu**	◯	◯	◯	◯
**3,03 Ndi capafupi kwa mwamuna kuuza mkazi kuti awlio agwilitse nchito mphira za kondomu**	◯	◯	◯	◯
**3,04 Ndidziwa kuti nditha ukhala nazo mphamvu zolamulira kuti tigwilitse nchito mpira za kondomu nthawi zonse pomwe tikugonana**	◯	◯	◯	◯
**3,05 Ndi cinthu cobvuta kupeza thandizo ya chilezi ku ana acicepele**	◯	◯	◯	◯
**3,06 Ndi cinthu capafupi ku ana acipele omwe ali mu chikwati kupeza thandizo ya chilezi**	◯	◯	◯	◯
**3,07 Ndingasankhe pa ine ndekha munthu amene ndifuna kumagonana naye**	◯	◯	◯	◯
**3,08 Makolo anga kapena achibululu ndiye ali nazo mphamvu zondisankhira munthu amene ndidzakwatilana naye**	◯	◯	◯	◯

3,09 Kodi munagwilitsako kale nchito mathandizo omwe apelekedwa mdela lanu ngati awa?

Read all services and select the ones used

- [ ] Chipatalachopima azimai ali ndipathupi/pakati/mimba ndi chipatala cha azimai pambuyo pobeleka mwana
- [ ] Mathandizo a chilezi
- [ ] Kupimitsa magazi kozisankhila inu nokha ndi colinga cofuna kudziwa ngati muli ndi kalombo ka HIV
- [ ] Mai kuteteza mwana wake ku kalombo ka HIV pamene mwanayo sanabadwe, panthawi yobeleka mwanayo kapena poyamwitsa mwana (PMTCT)
- [ ] Kuchotsa pathupi/pakati/mimba
- [ ] Kuthandizidwa pambuyo pochotsa pathupi/pakati/mimba
- [ ] Maphunziro ya modzisamalira ndi mauphungu pa nkhani za umuna ndi ukazi
- [ ] Zigawo zomwe zigwala nchito zoteteza ana acicepele monga ku polisi
- [ ] Nambala za malamya zotumidwa kwaulere pofuna kuthandizidwa
- [ ] Palibe mwamayanko apelekedwa
- [ ] Other

Specify other.

____

3,09z1 Kodi munagwilitsapo kale nchito njira ina yace iliyonse ya chilezi?

- ( ) Inde
- ( ) Ai

3,09z2 Kodi pali pano mulikugwiritsilako nchito njira yina iliyonse yacilezi?

- ( ) Inde
- ( ) Ai

» NDIME YA 4 - Nkhawa, ziyembekezo komanso ufulu wanga

Zotsatilazi zikuona pa maufulu ya ana acicepele maka-maka maufulu ya ana acikazi
Ndimada nkhawa ndi... . 0= Nditsutsa| 1= Ndibvomekezana nazo | SD = Sindidziwa | PY = Palibe yankho

	0	1	SD	PY
**4,01 Kukhala ndi pathupi kapena kupatsa pathupi mkazi ndikali mwana wacicepele**	○	○	○	○
**4,02 Kukhala mkwatibwi kapena mkwatili mofulumira ndikali mwana wacipele**	○	○	○	○
**4,03 Kulephela kutsiliza Sukulu**	○	○	○	○
**4,04 Kuyanganindwa cabe ngati mkwatibwi obweletsa malowolo/cimalo**	○	○	○	○
**4,04z Kuikidwa ngati mkwatili obweletsa chimalo cabe ngati nakwatila**	○	○	○	○
**4,05 Kusowa mpata/ufulu odzisankhila nekha munthu amene ndifuna kumayenda naye**	○	○	○	○
**4,06 Kukanidwa mpata wotenga mankhwala ya chilezi**	○	○	○	○

4,07 Kodi ceni-ceni comwe uganizira kawiri-kawiri pa umoyo wako ndi ciani?
Do not read the list, tick what is said

- ☐ Maphunziro/kutsiliza maphunziro yapamwamba
- ☐ Kupeza nchito/kusewenza/kutukuka
- ☐ Umoyo wanga/tsogolo yanga
- ☐ Kupeza/kumwa mankhwala
- ☐ Kalombo kamene kathetsa citetezo ca thupi la munthu (HIV)
- ☐ Kukhala ndi mnzanga wachikwati/Banja langa/ana anga
- ☐ Palibe yankho
- ☐ Other

Specify other.

4,08 Ngati mwana wacikazi wakhala ndi pathupi/pakati/mimba kuno kwanu, kodi angathamangile kwa yani kuti apeze thandizo?

Do not read the list, tick what is said

- [ ] Ku abanja/acibululu ena
- [ ] Anchito zaumoyo
- [ ] Aziphunzitsi
- [ ] Azikulu a mipingo
- [ ] Aphunzitsi achinyamata
- [ ] Mwamuna wake/bwenzi lacimuna/bwenzi lacikazi
- [ ] Anthu ena amaluso yakuya monga anchito za umoyo
- [ ] Palibe yankho
- [ ] Other

Specify other.

Kulinganiza zaimai ndi azibambo popanda kusankhulana

NDIME YA 5 - Kodi muganiza kuti zolemebedwa izi zili munsimu zikukhudzani motani inu:
0= Olo pan'gono pekha | 1= Pan'gono | 2 = Kothelatu | SD = Sindidziwa

	0	1	2	SD
**5,01 Ndimakhala ndimphamvu kulankhula ndi atsikana a munsinkhu wanga pa nkhani zokhuza ulinganiza kwa amuna ndi akazi ndi nkhani zokhuza ufulu wa ana acitsikana acicepele**	○	○	○	○
**5,02 Ndimakhala ndimphamvu kulankhula ndi anyamata a musinkhu wanga pa nkhani zokhuza ulinganiza kwa amuna ndi akazi ndi nkhani zokhuza ufulu wa ana acitsikana acicepele**	○	○	○	○
**5,03 Ndimakhala ndimphamvu kulankhula ndi azimai acikulire pa nkhani zokhuza ulinganiza kwa amuna ndi akazi ndi nkhani zokhuza ufulu wa ana acitsikana acicepele**	○	○	○	○
**5,04 Ndimakhala ndimphamvu kulankhula ndi azibambo acikulire pa nkhani zokhuza ulinganiza kwa amuna ndi akazi ndi nkhani zokhuza ufulu wa ana acitsikana acicepele**	○	○	○	○

Maukwati ya ana acicepele, kutengera pathupi kwa ana acicepele komanso mdulidwe wa ana acitsikana pomwe atha msinkhu

» NDIME YA 6 - Maukwati ya ana acicepele

6,01 Kodi muli pa ukwati kapena ai?

- ○ Mbeta/nkhungulume (Wosakwatira/wosakwatiliwa)
- ○ Mbeta/nkhungulume (wosakwatila/kukwatiliwa) koma ali ndi mwamuna wacisumbali
- ○ Mbeta/nkhungulume (wosakwatila/kukwatiliwa) koma ali ndi cisumbali cacikazi
- ○ Tinkhala pamodzi (koma sindise okwatilana)
- ○ Okwatiliwa na mwamuna umozi/mukazi umozi
- ○ Chipali
- ○ Chikwati cinatha
- ○ Ofedwa

- ◯ Anatayikana koma cikwati cikalibe kuthelatu

6,02 Kodi ndi njira zotani zodziwika bwino zomwe maukwati amacitikila mu dela lanu lino?
Read the list, tick all that apply

- ☐ Maukwati yopangika mocita kulembetsa kumalo olembetsera maukwati
- ☐ Maukwati ongopangika pakati pa mabanja awiri ya omwe akufuna kukwatilana
- ☐ Maukwati yolembetssedwa ku akulu olamulira dela monga amfumu
- ☐ Maukwati otsatira chipembedzo/mpingo - yolembetsa kumsikitini
- ☐ Maukwati otsatira chipembedzo/mpingo - yolembetsa kuchalichi
- ☐ Maukwati odziwika pambuyo pa banja la mkazi kulipila Chimalo
- ☐ Maukwati odziwika pambuyo pa banja la mwamuna kulipila Chimalo
- ☐ Other

Specify other.

____

» NDIME YA 7 - Pambuyo pa chikwati

7,01 Kodi ndipamsinkhu wotani pomwe munakwatilira koyamba?

____

7,02 Ndi liti kweni-kweni pamene inu munakwatilwa/kukwatira? (Lembani mwezi komanso caka)

mm/dd/yyyy

7,03 Kodi mnzanu amene munalowa naye mu ukwati anali ndi zaka zingati panthawi pamene munali kulowa naye mu ukwati?

____

7,04 Ngati mwayang'na kumbuyo, kodi muona kuti iyi inali nthawi yabwino komanso yoyenera kwa inu kuti mulowe mu ukwati?

Inde| Ai | SD = Sindidziwa | PY = Palibe yankho

- Inde
- Ai
- SD
- PY

7,05 Kodi cinali cinthu cozisankhila pa imwe nokha kuti mulowe mu ukwati panthawiyo?

Inde| Ai | SD = Sindidziwa | PY = Palibe yankho

- Inde
- Ai
- SD
- PY

7,06 Kodi munamvera ngati I munakakamizidwa ndi munthu wina wace kapena munthu wina wabanja lanu kuti mulowe mu ukwati?

Inde| Ai | SD = Sindidziwa | PY = Palibe yankho

- Inde
- Ai
- SD
- PY

7,07 Kodi munamvera ngati panali mabvuto ena yam'banja lanu kapena yamdela lanu yomwe yanapangitsa kuti mulowe m'banja panthawiyo?

Inde| Ai | SD = Sindidziwa | PY = Palibe yankho

- Inde
- Ai
- SD

- PY

7,08 Kodi mnzanu amene muli naye mu ukwati anampwetekankoni kapena kukumenyankoni kale olo tsopano?

Read the list, tick one

- Nthawi zonse
- Kawri-kawiri
- Nthawi zina
- Kulibe
- Sizimatero
- Sindifuna kuwulura pa izi

7,09 Kodi nkawiri-kawiri motani pomwe mumachitidwa cipongwe (monga ugwiliwa munjila yosayenera) pokhala kuti ndinu mkazi kapena mwamuna?

Read the list, tick one

- Tsiku ndi tsiku
- Kamodzi kapena kawiri pasabata imodzi
- Kamodzi kapena kawirir pa mwezi
- Kucepekera kamodzi pamwezi
- Sizimaterosizimachitika
- Sindifuna kuwulura pa izi

7,10 Kodi chikwati canu coyamba cinali colembetsedwa ku malo komwe kumalembetsedwera maukwati?

Read the list, tick all that apply

- [ ] Cosalembetsa
- [ ] Colembetsa kumpingo/ chikwati camwambo
- [ ] Cholembetsedwa kuchigawo ca Boma

7,11 Kodi muli naco chipepala comwe munalembetselapo ukwati wanu apa?

Inde| Ai | SD = Sindidziwa | PY = Palibe yankho

- Inde
- Ai
- SD
- PY

7,12 Pa maukwati onse a muno mdela lanu, ndinambala yotani yamaukwati yomwe yanamangidwa pamene wina ali ndi zaka zocepekera zaka khumi zisanu ndi zitatu (18)?

Read the list, tick one

- Akwatibwi onse
- Pafupi-fupi yonse
- Pafupi-fupi theka
- Pang'ono cabe
- Palibe
- Sindidziwa

7,13 Pa maukwati onse amene amapangika mdela lanu lino, kodi ndi nambala yamaukwati yangati yomwe yamangidwa mopanda mwini wake kubvomeleza? (mtsikana amene akwatiliwa)

Read the list, tick one

- Akwatibwi onse
- Pafupi-fupi yonse
- Pafupi-fupi theka
- Pang'ono cabe
- Palibe
- Sindidziwa

7,14 Pa waukwati onse amene amapangika mdela lanu lino, kodi ndi nambala yamaukwati yangati yomwe yamangidwa mopanda mwini wake kubvomeleza? (mzibambo amene akwatila)

Read the list, tick one

- Akwatibwi onse
- Pafupi-fupi yonse
- Pafupi-fupi theka
- Pang'ono cabe
- Palibe
- Sindidziwa

7,15 Pa waukwati onse amene amapangika mdela lanu lino, kodi ndi nambala yamaukwati yangati yomwe yamangidwa pamgwilizano wolipa ndalama, kapena mphatso zina zilizonse? (Mwacitsanzo kulipira chimalo)

Read the list, tick one

- Akwatibwi onse
- Pafupi-fupi yonse
- Pafupi-fupi theka
- Pang'ono cabe
- Palibe
- Sindidziwa

» NDIME YA 8 - Munthu akalibe kukwatila kapena kukwatiliwa

8,01 Ngati nkotheka, mufuna kuti mukakwatire muli ndi zaka zingati? (Nambala)

- Zaka
- Sindifuna kukwatira
- Palibe msinkhu weni-weni

8,02 Zaka

8,02 Zaka

8,03 Kodi mungafune kuonana ndi kudandaulirana ndi yani ngati mwaona kuti munthu wina wace ali kukukakamizani kuti mulowe mu ukwati popanda inu kufuna kutero?

- ☐ Amai/Atate
- ☐ Mbala wange
- ☐ Amalume
- ☐ Mlongo wamakolo anga wa mukazi
- ☐ Ambuya akazi kapena agogo amuna
- ☐ Aphunzitsi
- ☐ Amfumu
- ☐ Akulu ampingo
- ☐ Ku kalabu ya achinyamata
- ☐ Bwenzi langa
- ☐ Other

Specify other.

8,04 Pakati pa anthu okwatila muno mdela lanu, ndinambala yotani yamaukwati yomwe yanamangidwa mpamene wina ali ndi zaka zocepekera zaka khumi zisanu ndi zitatu (18)?

Read the list, tick one

- ◯ Akwatibwi onse
- ◯ Pafupi-fupi yonse
- ◯ Pafupi-fupi theka
- ◯ Pang'ono cabe
- ◯ Palibe

- Sindidziwa

8,05 Pamwaukwati onse amene amapangika mdela lanu lino, kodi ndi nambala yamaukwati yangati yomwe yamangidwa mopanda mwini wake kubvomeleza? (mtsikana amene akwatiliwa)

Read the list, tick one

- Akwatibwi onse
- Pafupi-fupi yonse
- Pafupi-fupi theka
- Pang'ono cabe
- Palibe
- Sindidziwa

8,06 Pamwaukwati onse amene amapangika mdela lanu lino, kodi ndi nambala yamaukwati yangati yomwe yamangidwa mopanda mwini wake kubvomeleza? (mzibambo amene akwatila)

Read the list, tick one

- Akwatibwi onse
- Pafupi-fupi yonse
- Pafupi-fupi theka
- Pang'ono cabe
- Palibe
- Sindidziwa

8,07 Pamwaukwati onse amene amapangika mdela lanu lino, kodi ndi nambala yamaukwati yangati yomwe yamangidwa pamgwilizano wolipa ndalama, kapena mphatso zina zilizonse? (Mwacitsanzo kulipira chimalo)

Read the list, tick one

- Akwatibwi onse
- Pafupi-fupi yonse
- Pafupi-fupi theka
- Pang'ono cabe
- Palibe
- Sindidziwa

8,08 Kodi nkawiri-kawiri motani pomwe mumacitidwa chipongwe pokhala ngati mkazi kapena mwamuna komanso kunenedwa?

Read the list, tick one

- Tsiku ndi tsiku
- Kamodzi kapena kawiri pasabata imodzi
- Kamodzi kapena kawirir pa mwezi
- Kucepekera kamodzi pamwezi
- Sizimaterosizimachitika
- Sindifuna kuwulura pa izi

» NDIME YA 9 - Kutenga pathupi kwa ana acicepele

9,01 Kodi muli ndi mwana kapena munakhalapo kale ndi mwana?

- Inde
- Ai

9,02 Kodi mwana wanu woyamba anabadwa liti? (Mwezi komanso caka)

mm/dd/yyyy

9,03 Kodi munakhalapo ndi mamimba angati/pakati kangati?

____

9,04 Kodi ndi ana angati pakati pa ana anu omwe akhala ndi inu panyumba panu?

____

9,05 Kodi munali ndi zaka zingati pamene munatenga mimba yoyamba ?

____

9,06 Kodi munali ndi msinkhu wotani pamene munakhala tate wa mwana?

____

9,07 Kodi cinali codzifunira kuti mukhale kholo panthawiyo?
Inde| Ai | SD = Sindidziwa | PY = Palibe yankho

- ○ Inde
- ○ Ai
- ○ SD
- ○ PY

9,08 Kodi ndi atsikana angati amene inu mudziwako amene anatenga mimba/pakati/pathupi pamene anali asanakwanitse zaka khumi zisanu ndi zitatu (18)?

____

9,09 Ndi njira yotani ya chilezi yomwe mukugwilitsa nchito?
Do not read the list, tick what is said

- [ ] Palibe
- [ ] Chilezi cacilengedwe
- [ ] Mphira za kondomu
- [ ] Kondomu za yacikazi
- [ ] Njira yacilezi yolowetsa mu ukazi kuletsa mphamvu ya umuna kupita
- [ ] Mapilisi ya chilezi
- [ ] Chilezi cansingano
- [ ] Chilezi colowetsa pamwamba padzanja la mkazi
- [ ] Kacida koteteza mimba kocita kulowetsa mcibaliro
- [ ] Kuthetsa mphamvu yamwamuna
- [ ] Sindidziwa
- [ ] Other

Specify other.

____

9,10 Kodi ndi njira zacilezi zotani zomwe nza pafupi kupeza mdela lanu lino?

Do not read the list, tick what is said

- ☐ Palibe
- ☐ Chilezi cacilengedwe
- ☐ Mphira za kondomu
- ☐ Kondomu za yacikazi
- ☐ Njira yacilezi yolowetsa mu ukazi kuletsa mphamvu ya umuna kupita
- ☐ Mapilisi ya chilezi
- ☐ Chilezi cansingano
- ☐ Chilezi colowetsa pamwamba padzanja la mkazi
- ☐ Kacida koteteza mimba kocita kulowetsa mcibaliro
- ☐ Kuthetsa mphamvu yamwamuna
- ☐ Sindidziwa
- ☐ Other

Specify other.

____

9,11 Kodi ndi nthawi zotani pamene munthu angapeze njira za makono za chilezi? (Njira iliyonse yachilezi kuchotserapo njira ya chilezi ca chilengedwe)

Read the list, tick which one is said

- ◯ Nthawi zonse
- ◯ Nkosatheka
- ◯ Pokhapo ngati ali mu ukwati
- ◯ Ngati wayamba kubeleka ana
- ◯ Pamene watha msinkhu
- ◯ Pamsinkhu wina wace

9,12 Zaka

____

» NDIME YA 10 - Maukwati ya ana: mwambo, zikhulipiliro komanso makhalidwe

10,01 Mumaganizo anu kodi ndi pamsinkhu wotani pamene mwana WACIKAZI mungakambe kuti ndi wamkulu?

____

10,02 Mumaganizo anu kodi ndi pamsinkhu wotani pamene mwana WACIMUNA mungakambe kuti ndi wamkulu?

____

10,03 Kambiri-kambiri mdela lanu lino kodi AZIMAI amakhala ali ndi moyo kwanthawi itali bwanji? (zaka zokhala ndi moyo)

____

10,04 Kambiri-kambiri mdela lanu lino kodi AZIBAMBO amakhala ali ndi moyo kwanthawi itali bwanji? (zaka zokhala ndi moyo)

____

10,05 Mumaganizo anu ndi msinkhu wotani omwe ndiwoyenelera kuti mwana WAMKAZI akwatiliwe?

____

10,06 Mumaganizo anu ndi msinkhu wotani omwe ndiwoyenelera kuti mwana WACIMUNA akwatire?

10,07 Mumaganizo anu kodi ndi pa msinkhu waukulu motani pomwe mungakambe kuti MKAZI wakalamba kwambiri (kopita malire) kuti akwatiliwe?

10,08 Mumaganizo anu kodi ndi pa msinkhu waukulu motani pomwe mungakambe kuti MWAMUNA wakalamba kwambiri (kopita malire) kuti akwatiliwe?

10,09 Mumaganizo anu kodi ndi pa msinkhu waukulu motani pomwe mungakambe kuti MKAZI wacepa kwambiri (kopita malire) kuti akwatiliwe?

10,10 Mumaganizo anu kodi ndi pa msinkhu waukulu motani pomwe mungakambe kuti MWAMUNA wacepa kwambiri (kopita malire) kuti akwatiliwe?

Kodi, mugwirizana nazo bwanji zamene zili mundondomeko iyi?

0= Ndikanitsitsa| 1= Ndikana | 2= Ndikanako pang'ono | 3= Zilipakatimpakati | 4= Ndizoonako | 5= Ndizoona | 6= Ndizoona kwambiri | SD= Sindidziwa

	0	1	2	3	4	5	6	SD
**10,11 MTSIKANA ali ofikapo kukwatiliwa ngati wangoyamba kupita kumwezi**	○	○	○	○	○	○	○	○
**10,12 Pali ubwino waukulu ngati MTSIKANA wakwatiriwa pamene zaka zakubadwa zisanafike khumi, zisanu ndi zitatu (18)**	○	○	○	○	○	○	○	○
**10,13 MNYAMATA?**	○	○	○	○	○	○	○	○
**10,14 Pali kuipa kwace ngati MTSIKANA akwatiliwa pamene zaka zakubadwa zisanafike khumi zisanu ndi zitatu (18)**	○	○	○	○	○	○	○	○
**10,15 MNYAMATA?**	○	○	○	○	○	○	○	○
**10,16 Kukwatilitsa mwana WACIKAZI akalibe ufika zaka khumi zisanu ndi zitatu ndi mwambo komanso mkhalidwe wathu**	○	○	○	○	○	○	○	○
**10,17 WACIMUNA?**	○	○	○	○	○	○	○	○

Kodi, mugwirizana nazo bwanji zamene zili mundondomeko iyi?

0= Ndikanitsitsa| 1= Ndikana | 2= Ndikanako pang'ono | 3= Zilipakatimpakati | 4= Ndizoonako | 5= Ndizoona | 6= Ndizoona kwambiri | SD= Sindidziwa

	0	1	2	3	4	5	6	SD
**10,18 Kukwatilitsa mwana WACIKAZI akali wa cicepele ndi mwambo wa mpingo wathu/chipembedzo cathu**	◯	◯	◯	◯	◯	◯	◯	◯
**10,19 WACIMUNA?**	◯	◯	◯	◯	◯	◯	◯	◯
**10,20 Kukwatilitsa mwana WACIKAZI akali wacicepele cithandiza kuti banja lamtsikanayo likhale lolemekezeka ndi laulemu**	◯	◯	◯	◯	◯	◯	◯	◯
**10,21 WACIMUNA?**	◯	◯	◯	◯	◯	◯	◯	◯
**10,22 Kukwatilitsa mwana WACIKAZI akali wacicepele amene akalibe kuwanilitsa zaka khumi zisanu ndi zitatu (18) zingacitike mdela lathu lino ngati mwanayo watenga pathupi/pakati/mimba**	◯	◯	◯	◯	◯	◯	◯	◯
**10,23 WACIMUNA?**	◯	◯	◯	◯	◯	◯	◯	◯

Kodi, mugwirizana nazo bwanji zamene zili mundondomeko iyi?

0= Ndikanitsitsa| 1= Ndikana | 2= Ndikanako pang'ono | 3= Zilipakatimpakati | 4= Ndizoonako | 5= Ndizoona | 6= Ndizoona kwambiri | SD= Sindidziwa

	0	1	2	3	4	5	6	SD
**10,24 Kukwatilitsa mwana WACIKAZI amene akalibe kukwanilitsa zaka khumi, zisanu ndi zitatu kungacitike ncolinga cofuna kuthetsa mkangano wam'banja**	◯	◯	◯	◯	◯	◯	◯	◯
**10,25 WACIMUNA?**	◯	◯	◯	◯	◯	◯	◯	◯
**10,26 Ana ACITSIKANA amene abeleka pakati pa zaka khumi ndi zisanu kufikira pa zaka kuhumi, zisanu ndi zitatu (15 – 18) kambirikambiri amepezeka kuti amakhala ndi mimba yathanzi ndiponso amabeleka ana athanzi labwino (kupambana atsikana a zaka khumi, zisanu ndi zitatu - 18), cifukwa cakuti mathupi awo amakhala akali aciceple ndiponso akali athanzi ndi olipa kwambiri**	◯	◯	◯	◯	◯	◯	◯	◯
**10,27 Maukwati ya ana ACIKAZI omwe amacitika pamene mtsikana akalibe kukwanilitsa zaka khumi zisanu ndi zitatu (18) amacitika cifukwa ca cikondi**	◯	◯	◯	◯	◯	◯	◯	◯

**10,28 ACIMUNA?**	◯	◯	◯	◯	◯	◯	◯	◯

Kodi, mugwirizana nazo bwanji zamene zili mundondomeko iyi?

0= Ndikanitsitsa| 1= Ndikana | 2= Ndikanako pang'ono | 3= Zilipakatimpakati | 4= Ndizoonako | 5= Ndizoona | 6= Ndizoona kwambiri | SD= Sindidziwa

	0	1	2	3	4	5	6	SD
**10,29 Maukwati ya ana ACIKAZI azaka zocepekela pa khumi zisanu ndi zitatu (18) amacitika cifukwa cosowekera maphunziro ndiponso kusowekera kwa umwayi wopeza zinchito**	◯	◯	◯	◯	◯	◯	◯	◯
**10,30 ACIMUNA?**	◯	◯	◯	◯	◯	◯	◯	◯
**10,31 Kukwatilitsa mwana WACIKAZI amene akalibe kufikitsa zaka khumi zisanu ndi zitatu (18) kungathandize kuthetsa mabvuto ya za chuma (ndalama) m'banja**	◯	◯	◯	◯	◯	◯	◯	◯
**10,32 WACIMUNA?**	◯	◯	◯	◯	◯	◯	◯	◯
**10,33 Kukwatilitsa mwana WACIKAZI amene akalibe kukwanilitsa zaka khumi zisanu ndi zitatu (18) kuthandiza ngati njira yopelekeramo citetezo ku mwanayo**	◯	◯	◯	◯	◯	◯	◯	◯
**10,34 WACIMUNA?**	◯	◯	◯	◯	◯	◯	◯	◯

Kodi, mugwirizana nazo bwanji zamene zili mundondomeko iyi?

0= Ndikanitsitsa| 1= Ndikana | 2= Ndikanako pang'ono | 3= Zilipakatimpakati | 4= Ndizoonako | 5= Ndizoona | 6= Ndizoona kwambiri | SD= Sindidziwa

	0	1	2	3	4	5	6	SD
**10,35 Mkwatibwi wacicepele kambili amafunika ulipilidwa ndalama ya chimalo yocepekera kuposana ndi mkwatidwi wacikulire**	○	○	○	○	○	○	○	○
**10,36 Kukwatilitsa mwana WACIKAZI akalibe ukwanitsa zaka khumi zisanu ndi zitatu (18) nthawi zina kucitika kamba ka nkhani za chuma (ndalama)**	○	○	○	○	○	○	○	○
**10,37 WACIMUNA?**	○	○	○	○	○	○	○	○
**10,38 Kukwatilitsa mwana WACIKAZI akali wacicepele kuthandiza kuti mwanayo asamagonedwe mwacikakamizo, kupwetekedwa komanso kuputidwa**	○	○	○	○	○	○	○	○
**10,39 WAMWAMUNA?**	○	○	○	○	○	○	○	○
**10,40 Ana ACITSIKANA amene atenga pathupi akalibe ufika pa msinkhu wa zaka khumi, zisanu ndi zitatu (18) atha kupezana ndi mabvuto yambiri yaumoyo kapena usakhala wa thanzi ndiponso atha kupita mwacabe kusiyana ndi atsikana opitilira zaka khumi zisanu ndi ziitatu (>18)**	○	○	○	○	○	○	○	○

Kodi, mugwirizana nazo bwanji zamene zili mundondomeko iyi?

0= Ndikanitsitsa| 1= Ndikana | 2= Ndikanako pang'ono | 3= Zilipakatimpakati | 4= Ndizoonako | 5= Ndizoona | 6= Ndizoona kwambiri | SD= Sindidziwa

	0	1	2	3	4	5	6	SD
**10,41 Kukwatilitsa mwana WACIKAZI WACITSIKANA amene akalibe ufika pa msinkhu wa zaka khumi zisanu ndi zitatu (18) kumapangitsa kuti mtsikanao akhale ndi mabvuto pa nkhani yamaphunziro**	○	○	○	○	○	○	○	○
**10,42 WACIMUNA?**	○	○	○	○	○	○	○	○
**10,43 Kukwatilitsa mwana WACIKAZI akali wacicepele nkwabwino kwambiri cifukwa amakhala omvela ndi waulemu kwambiri ku mwamuna wake**	○	○	○	○	○	○	○	○
**10,44 Angakhale kuti mwana WACIKAZI safuna kukwatiliwa iye mwini, afunikira kulemekeza ciganizo comwe makolo ake apanga ndiponso kulemekeza zofuna zabanja lake**	○	○	○	○	○	○	○	○
**10,45 WACIMUNA?**	○	○	○	○	○	○	○	○
**10,46 Nthawi zina nkofunika kwambiri kumenya mwana WACIKAZI komanso kupeleka cilango colimba kwambiri ngati mwanayo wabweletsa musebanya kubanja lake olo kapena safuna kulondola zomwe banja lake li kufuna**	○	○	○	○	○	○	○	○
**10,46z Nthawi zina nkofunika kwambiri kumenya mwana WACIMUNA komanso kupeleka cilango colimba kwambiri ngati mwanayo wabweletsa musebanya ku banje lake olo kapena safuna kulondola zomwe banja lake ili kufuna**	○	○	○	○	○	○	○	○

Kodi, mugwirizana nazo bwanji zamene zili mundondomeko iyi?

0= Ndikanitsitsa| 1= Ndikana | 2= Ndikanako pang'ono | 3= Zilipakatimpakati | 4= Ndizoonako | 5= Ndizoona | 6= Ndizoona kwambiri | SD= Sindidziwa

	0	1	2	3	4	5	6	SD
**10,47 Mwana WACITSIKANA afunika kupatsidwa mpata wodzisankhila mwamuna amene iye mwini afuna kukwatiliwa naye**	○	○	○	○	○	○	○	○
**10,48 Mwana WACIMUNA afunika kupatsidwa mpata wodzisankhila mkazi amene iye mwini afuna kukwatirana naye**	○	○	○	○	○	○	○	○
**10,49 Mwana WACITSIKANA safunika kukakamizidwa kuti akwatiliwe**	○	○	○	○	○	○	○	○
**10,50 WACIMUNA?**	○	○	○	○	○	○	○	○
**10,51 MUKAZI WOKWATIWA afunika kulemekeza ndikumvera zokamba zamwamuna wake nthawi zonse**	○	○	○	○	○	○	○	○
**10,52 AMUNA afunika ukhala atsogoleri a banja lonse**	○	○	○	○	○	○	○	○
**10,53 MWAMUNA afunika kukwatira mkazi m'modzi cabe**	○	○	○	○	○	○	○	○
**10,54 Ana ACITSIKANA amene amakwatilitsidwa pamene akalibe kukwanilitsa zaka khumi zisanu ndi zitatu (18) nthawi zambiri amakwatilitsa ku mwamuna amene ali kale ndi mkazi kapena azikazi ena**	○	○	○	○	○	○	○	○

» NDIME YA 11 - Malamulo okhazikitsidwa omwe akamba pa nkhani za maukwati

11,01 Kodi mukudziwa kuti pali lamulo yolembedwa mdziko muno yomwe ifotokoza msinkhu woyenelera kuti munthu akhale kapena kuti alowe muchikwati?

Inde| Ai | SD = Sindidziwa | PY = Palibe yankho

- ○ Inde
- ○ Ai
- ○ SD
- ○ PY

11,02 Kodi ndipamsinkhu wotani pomwe lamulo yolembedwa ya dziko lino ibvomelezerapo kuti munthu alowe mu chikwati? Kuli atsikana

11,03 Kodi ndipamsinkhu wotani pomwe lamulo yolembedwa ya dziko lino ibvomelezerapo kuti munthu alowe mu chikwati? Kuli anyamata

11,04 Kodi ana acicepele abvomelezedwa kulowa muchikwati asanafike pa musinkhu oledwa mwa lamulo?
Read the list, tick which one is said

- ◯ Nthawi zonse
- ◯ Kawri-kawiri
- ◯ Nthawi zina
- ◯ Kulibe
- ◯ Sizimatero
- ◯ Sindifuna kuwulura pa izi

11,05 Kodi pali wina aliyense amene amalankhulapo ngati mwana walowa muchikwati kapena pamene akufuna kulowa muchikwati akalibe ufika pa msinkhu ololedwa?
Inde| Ai | SD = Sindidziwa | PY = Palibe yankho

- ◯ Inde
- ◯ Ai
- ◯ SD
- ◯ PY

11,06 Kodi amene amalankhulapo kawiri-kawiri pa izi ndani?
Do not read the list, tick all that apply

- ☐ APolisi
- ☐ Opititsa patsogolo kasamaliro kamalamulo
- ☐ Akulu olamulira ku dela (mwacitsanzo: amene amanveledwa kwambiri ngati alankhula zinthu')
- ☐ Anyanchito ocokera kumabungwe ya za citukuko kapena ma bungwe odziimila pa okha (NGO)

☐ Other

Specify other.

____

11,07 Ndi pamuyeso wotani womwe amalankhulirapo zomwe izi zitacitika?
Read the list, tick which one is said

- ○ Nthawi zonse
- ○ Kawri-kawiri
- ○ Nthawi zina
- ○ Kulibe
- ○ Sizimatero
- ○ Sindifuna kuwulura pa izi

11,08 Kodi ndi msinkhu wotani womwe ndiwobvomekezeka kuti munthu alowe mu ukwati kulingana ndi malamulo a mpingo wanu kapena chipembedzo canu? AKAZI
Read the list, tick which one is said

- ○ Palibe muyeso wa msinkhu
- ○ Pamene watha msinkhu
- ○ Msinkhu (nambala)
- ○ Sindidziwa

11,09 Zaka

____

11,10 Kodi ndi msinkhu wotani womwe ndiwobvomekezeka kuti munthu alowe mu ukwati kulingana ndi malamulo a mpingo wanu kapena chipembedzo canu? AMUNA
Read the list, tick which one is said

- ○ Palibe muyeso wa msinkhu
- ○ Pamene watha msinkhu
- ○ Msinkhu (nambala)

- Sindidziwa

11,11 Zaka

____

11,11z1 Kodi ndi msinkhu wotani womwe ndiwobvomekezeka kuti munthu alowe mu ukwati kulingana ndi malamulo amtundu wanu? AKAZI

Read the list, tick which one is said

- Palibe muyeso wa msinkhu
- Pamene watha msinkhu
- Msinkhu (nambala)
- Sindidziwa

11,11z2 Zaka

____

11,11z3 Kodi ndi msinkhu wotani womwe ndiwobvomekezeka kuti munthu alowe mu ukwati kulingana ndi malamulo amtundu wanu? AMUNA

Read the list, tick which one is said

- Palibe muyeso wa msinkhu
- Pamene watha msinkhu
- Msinkhu (nambala)
- Sindidziwa

11,11z4 Zaka

____

11,12 Kodi pali cili conse comwe mungakonde kuti mutiuzeko panthawi ino?

____

» NDIME YA 12 - Maganizo ya pa nkhani ya maukwati ya ana

Kodi inuyo muganizapo bwanji pa nkhani yokhudza maukwati ya ana acicepele?
Inde| Ai | SD = Sindidziwa | PY = Palibe yankho

	Inde	Ai	SD	PY
**12,01 Phindu imakhala ya anthu a mu banja osati kumwanayo**	◯	◯	◯	◯
**12,02 Ndiye yankho yokhayo ngati mwana wacitsikana watenga pathupi/mimba/pakati**	◯	◯	◯	◯
**12,04 Zimacitika kawiri-kawiri pokhapo ngati mwana wacitsikana watenga pathupi/mimba/pakati**	◯	◯	◯	◯
**12,05 Ndidziwa za ana acitsikana ambiri amene akwatilitsidwa popanda iwo ene ake kufuna kulowa mu chikwati**	◯	◯	◯	◯
**12,06 Ndidziwa mopewela kutenga pathupi/mimba/pakati**	◯	◯	◯	◯
**12,07 Ngati mtsikana walowa muchikwati amasiya kupita kusukulu**	◯	◯	◯	◯
**12,08 Ngati ana acitsikana ndi acinyamata asiya kupita kupita kusukulu, amasowelekera mauthenga yoyenelera**	◯	◯	◯	◯
**12,09 Njira yokhayo yomwe ingathandize kuthetsa maukwati ya ana acicepele ndikuthandizira anawo kuti akhale ndi maziko eni-eni a chuma**	◯	◯	◯	◯
**12,10 Anyamata ndi azibambo afunika kugwilizana ndi kugwapo mofikapo pakufuna kuthetsa bvuto ya maukwati ya ana a cicepele**	◯	◯	◯	◯

ZIKOMO KWAMBIRI CHIFUKWA CA NTHAWI YANU YOMWE MUNATIPSA KULANKHULANA NANU

Any questions?

14,01 NDIME YA 14 - Kodi pangakhale funso yomwe mufuna kutifunsa?

____

Interviewer's comments

15,01 NDIME YA 15 - NDEMANGA ZOCOKERA KU MUNTHU AMENE ANALI KUFUNSA MAFUNSO

____

000 Please register the location

[illegible]

latitude (x.y °)	longitude (x.y °)	altitude (m)	accuracy (m)
